# The effect of COVID-19 pandemic on diabetes care indices in Southern Iran: an interrupted time series analysis

**DOI:** 10.1186/s12913-023-09158-4

**Published:** 2023-02-13

**Authors:** Alireza Mirahmadizadeh, Mohammad Hossein Sharifi, Jafar Hassanzadeh, Alireza Heiran, Fariba Moradi Ardekani, Neda Hadizadeh, Mehdi Sharafi, Mohammad Mohammadi Abnavi

**Affiliations:** 1 Non-Communicable Diseases Research Center, Shiraz University of Medical Sciences, Shiraz, Iran; 2 Research Center for Health Sciences, Institute of Health, School of Health, Department of Epidemiology, Shiraz University of Medical Sciences, Shiraz, Iran; 3 Non-Communicable Diseases Group Manager, Office of Vice Chancellor for Health Affairs, Shiraz University of Medical Sciences, Shiraz, Iran; 4 Student Research Committee, Shiraz University of Medical Sciences, School of Health, Shiraz, Iran; 5 Noncommunicable Diseases Research Center, Fasa University of Medical Sciences, Fasa, Iran

**Keywords:** Type 2 diabetes, COVID-19, Diabetes care

## Abstract

**Background:**

Type 2 diabetes mellitus (T2DM) requires a continues bulk of cares. It is very probable COVID-19 pandemic is affected its healthcare coverage.

**Methods:**

The interrupted time series analysis is used to model the trend of diabetes healthcare indices, such as the health worker visits, physician visits, body mass index (MBI), fasting blood sugar (FBS), and hemoglobin A1c (HbA1c), before and after the start of COVID-19 pandemic. The reference of data was the totals of all T2DM patients living in Fars Province, Southern Iran, areas covered by Shiraz University of Medical Science (SUMS), from 2019 to 2020.

**Results:**

A significant decrease for visits by the health workers, and physicians was observed by starting COVID-19 pandemic (β_2_ = -0.808, *P* < 0.001, β_2_ = -0.560, *P* < 0.001); Nevertheless, the coverage of these services statistically increased by next months (β_3_ = 0.112, *P* < 0.001, β_3_ = 0.053, *P* < 0.001). A same pattern was observed for the number of BMI, FBS and HbA1c assessments, and number of refer to hospital emergency wards (β_3_ = 0.105, *P* < 0.001; β_3_ = 0.076, *P* < 0.001; β_3_ = 0.022, *P* < 0.001; β_3_ = 0.106, *P* < 0.001). The proportion of T2DM patients with HbA1C < 7%, and controlled hypertension during study period was statistically unchanged.

**Conclusions:**

When the COVID-19 pandemic was announced, T2DM healthcare coverage drastically decreased, but it quickly began to rebound. The health monitoring system could not have any noticeable effects on diabetes outcomes.

**Supplementary Information:**

The online version contains supplementary material available at 10.1186/s12913-023-09158-4.

## Introduction

Diabetes is one of the most prevalent chronic disorders that has reached the epidemic proportions worldwide, exerting a substantial burden on the health care service [1]. Based on the estimates from 2019, 1.1 million children and 463 million adults (aged 20 to 79) worldwide have diabetes, and it is expected to increase to 700 million by 2045 [2]. Furthermore, 12% of Iranian individuals have diabetes, placing a significant strain on country's healthcare system [3].

Like many chronic diseases, the patients with type 2 diabetes mellitus (T2DM) need a long-standing healthcare coverage to prevent micro- and macro-vascular complications [4]. The coronavirus disease 2019 (COVID-19), the most recent infectious disease to go global, was shown to have a common underlying illness across COVID-19 patients that has a discernible effect on the disease's course and prognosis [5–8]. Besides, a new pandemic – i.e., COVID-19 – affects healthcare system framework in response to the influx of the patients with COVID-19, including the divergence of resources, and bias toward the disease itself, the redistribution of health services infrastructures (health workers, equipment, and facilities), financial barriers, physical restrictions, and public fear of infection. By these inescapable challenges, governments, especially low-to-middle income countries, have faced declines in the health service coverage. Consequently, by emerging these direct and indirect effects, diabetes healthcare coverage might drop dramatically [9], which might increase the burden of diabetes. Hence, it is essential to study the changes in diabetes healthcare indices to find solutions to improve the diabetes care.

People who suffer from the chronic conditions are more prone to infections. Such factors decrease the likelihood of the recovery for COVID-19 patients [10, 11]. The most difficult aspect of a pandemic is continuing to provide normal treatment for chronic conditions [12]. The difficulties of the routine healthcare provision for T2DM patients during COVID-19 pandemic in Iran could be summarized in nine themes as follows: the lockdown of standard outpatient clinics, decreased inpatient capacity, staff shortage, medical facilities shortage, unaffordable medicine, delayed care seeking, limited self-care practice, transport difficulties, and undiagnosed cases/events [13]. During the study period, there was a lockdown for COVID-19 in Iran. We experienced 3 peaks of COVID-19 pandemic. Moreover, the significant changes were observed in the diabetes self-management behaviors, and using the telemedicine and virtual and online visits increased [14, 15].

Some evidence indicates serious damages to cover the chronic diseases’, especially diabetes, preventive and therapeutic services during current pandemic [16–19]. While in another study, there was no difference between the control and quarantine groups due to body mass index (BMI), plasma glucose, or hemoglobin A1c (HbA1c) levels [20]. However, the supporting data is weak, and no comparable research using Iranian data was performed. We sought to assess the effect of COVID-19 pandemic on diabetes healthcare indices in the health centers under the auspices of Shiraz University of Medical Sciences, Shiraz, southern Iran.

## Methods

In this ecologic study, we assessed the effect of COVID-19 announcement as a natural intervention to T2DM patients’ healthcare indices. From January 01, 2020 to January 01, 2021, 160,000 T2DM patients who were under cover of Shiraz University of Medical Sciences, Shiraz, Iran in health centers of 29 counties in Fars province, included into the study. The population of Fars province was stable before and after the epidemic.

The target statistics were the totals of all T2DM patients living in the Fars Province, Southern Iran, areas covered by the Shiraz University of Medical Science (SUMS), from 2019 to 2020.As a nature of administrative data, in each visit, diabetes care parameters were electronically recorded in the Integrated Health System (in Persian: “Samaneh Yekparche-ye Behdashti”) by health staff mainly working in primary and secondary healthcare centers (including 29 cities, urban and rural centers). For research purpose, the seasonal aggregated counts for various indices were integrated in the SUMS Department of Health. Individual data were not collected.

The final dataset contained several aggregated variables, including numbers of the patients with T2DM and newly diagnosed patients with T2DM, sex, season, diabetes care-related measurements, such as health worker visits, physician visits, BMI (body mass index) [3 levels, desired level < 25 kg/m2], FBS (fasting blood sugar) [3 levels, desired level of 70–130 mg/dL], and HbA1c (hemoglobin A1c) [5 levels, desired level < 7%], health status and outcome variables, such as hypertension (BP < 140/90 mmHg), uncontrolled hypertension (BP > 140/90 mmHg), microvascular and macrovascular complications, emergency refers to hospital (e.g., sudden complication, dysglycemia, etc.), and the reasons (death, migration, etc.) do not go to the health centers.

Based on the national guideline [21], each T2DM patient should be regularly seen by a health care worker every month and a physician every three months. The health professionals should assess the patient's BMI and mean blood pressure at each visit and record the data every three months. Besides, family doctors should send patients to laboratory facilities to assess HbA1c every 6 and 3 months, respectively, for those with and without glycemic management. The primary result of our study was to assess the immediate secular trend, induced by COVID-19 pandemic announcement, on T2DM healthcare indices. The secondary outcome was to track the seasonal trend of these indices one year before, and after COVID-19 pandemic announcement, using Interrupted Time Series analysis.

The point intervention day was set as February 20, 2020 when the first case of COVID-19 officially announced in Shiraz, seat of Fars province. Statistical analysis was done using the STATA (Stata Corp (2017) Stata Statistical Software: Release 15. College Station, TX: Stata Corp LLC.). We used seasonal values as the unit of time period for interrupted time series analysis as well as aggregated data; hence we could not assess the heterogeneity. Besides, we assumed that the revelation of the COVID-19 pandemic was the only unexpected intervention or event that happened throughout the research period. To test this hypothesis, we performed a non-parametric correlation analysis (Spearman's rho) among three pandemic severity indicators (seasonal counts of the COVID-19 incidence rate, hospitalization rate, and fatality rate) and their corresponding seasonal counts of T2DM care indices. In general, we did not observe any correlation; that is, the T2DM healthcare services changes or recovery during the first year of COVID-19 pandemic did not potentially affected by the pandemic severity (table S18). For data handling, Microsoft Excel data analysis software was used. Data were described as mean (95% confidence interval (CI)). The segmented Poisson regression was selected since each dependent variable was constituted from the counts of care delivered in a season. The interrupted time series models require at least three independent variables [22]:

$$Yt = \beta 0 + \beta 1T + \beta 2Xt + \beta 3 (T - Ti) Xt$$

Beta (β) coefficients indicates trend or level of change in each variable, including β1 for the trend before COVID-19 pandemic, β2 for the early change in level induced by the COVID-19 natural intervention, and β3 for the trend of recovery (or deterioration) during COVID-19 pandemic. For each β coefficients, a P-value ≤ 0.05 indicates that a trend or change is statistically significant.

## Result

Approximately a mean 160,000 T2DM patients (61.9% female and 79.5% urban resident) were seasonally observed from 2019 to 2020. A mean of 6,254 individuals (55.7% female, 80.4% urban resident) were newly diagnosed with T2DM (Table 1). The mean prevalence of T2DM was 7.2%; and 6.9 and 7.3% in 2019 and 2020, respectively. Moreover, the mean incidence of T2DM was 30 per 10,000 people with 40.3 and 19.9 per 10,000 people in 2019 and 2020, respectively.

**Table 1:** The demographic information of the participants during 2019–2020

Year Index		2019				2020			
		Spring	Summer	Autumn	Winter	Spring	Summer	Autumn	Winter
**Total population [n]**	**Female**	2,039,733	2,085,864	2,088,745	2,089,043	2,077,130	2,128,471	2,128,443	2,128,443
	**Male**	2,018,263	2,071,013	2,072,303	2,071,402	2,065,557	2,123,073	2,123,086	2,126,611
	**Total**	4,057,996	4,156,877	4,161,048	4,160,445	4,142,687	4,251,544	4,251,529	4,255,054
	**Urban**	2,956,990	3,034,509	3,040,175	3,039,280	3,016,843	3,098,098	3,098,255	3,101,780
	**Rural**	1,101,006	1,122,368	1,120,873	1,121,165	1,125,844	1,153,446	1,153,274	1,153,274
**Population ≥ 30 years [n]**	**Female**	1,112,672	1,137,509	1,124,994	1,124,709	1,133,161	1,125,324	1,146,249	1,146,249
	**Male**	1,084,470	1,092,201	1,091,774	1,091,762	1,117,044	1,124,482	1,125,454	1,125,454
	**Total**	2,197,142	2,229,710	2,216,768	2,216,471	2,250,205	2,249,806	2,271,703	2,271,703
	**Urban**	1,640,504	1,672,772	1,663,569	1,663,834	1,689,420	1,698,649	1,719,653	1,719,653
	**Rural**	556,638	556,938	553,199	552,637	560,785	551,157	552,050	552,050
**T2DM population ≥ 30 years [n]**	**Female**	91,573	94,692	97,957	99,386	100,353	102,266	104,195	102,966
	**Male**	53,709	58,992	61,373	61,078	59,739	62,932	64,593	66,659
	**Total**	145,282	153,684	159,330	160,464	160,092	165,198	168,788	169,625
	**Urban**	115,477	121,633	126,426	127,942	127,856	132,393	133,312	134,688
	**Rural**	29,805	32,051	32,904	32,522	32,236	32,805	35,476	34,937
**New T2DM population ≥ 30 years [n]**	**Female**	4,731	4,938	4,958	3,839	1,320	4,390	2,037	1,674
	**Male**	3,469	3,815	4,115	3,461	709	2,958	1,478	2,143
	**Total**	8,200	8,753	9,073	7,300	2,029	7,348	3,515	3,817
	**Urban**	6,844	7,281	8,108	6,339	1,444	5,772	2,306	2,162
	**Rural**	1,356	1,472	965	961	585	1,576	1,209	1,655

*T2DM* Type 2 diabetes mellitus

The mean seasonal number of all diabetes care indices was decreased in 2020 comparing 2019, which are 49.9% vs. 35.8% in health worker visits, 43.9% and 28.9% in physician visits, 49.5% vs. 35.3% in BMI assessment, 38.7% vs. 25.7% in FBS assessment, and 23.9% vs. 14.1% in HbA1c assessment. This consistent reduction in the aforementioned indices was found for every pair of comparable seasons in 2019–2020 (Table 2). Besides, seasonal crude figures for the health condition of T2DM patients in 2020–2021 are included in Table 2.

**Table 2:** T2DM care numbers and health status values during 2020–2021

Year Index	2019				2020			
	Spring	Summer	Autumn	Winter	Spring	Summer	Autumn	Winter
Health worker visit	81,987 (56.4) ^a^	80,510 (52.4)	74,181 (46.6)	72,139 (45.0)	53,761 (33.6)	58,280 (35.3)	59,106 (35.0)	66,530 (39.2)
Physician visit	69,566 (47.9)	72,570 (47.2)	65,294 (41.0)	64,266 (40.1)	43,767 (27.3)	53,658 (32.5)	44,062 (26.1)	50,049 (29.5)
BMI assessment	80,367 (55.3)	80,251 (52.2)	73,607 (46.2)	72,151 (45.0)	53,314 (33.3)	57,440 (34.8)	57,584 (34.1)	65,955 (39.2)
FBS measurement	61,344 (42.2)	62,618 (40.7)	59,702 (37.5)	55,619 (34.7)	36,846 (23.0)	47,567 (28.8)	40,725 (24.1)	45,195 (26.8)
HbA1C measurement	37,458 (25.8)	37,184 (24.2)	38,408 (24.1)	35,043 (21.8)	20,601 (12.9)	27,162 (16.4)	22,785 (13.5)	22,508 (13.4)
BMI < 25 kg/m^2^	21,270 (26.5)	22,888 (28.5)	21,583 (29.3)	20,915 (29.0)	16,213 (30.4)	17,523 (30.5)	16,597 (28.8)	19,412 (29.4)
FBS 70–130 mg/dL	34,920 (56.9)	33,745 (53.9)	33,567 (56.2)	32,182 (57.9)	20,133 (54.6)	26,633 (56.0)	23,723 (58.3)	25,941 (57.4)
HbA1c < 7%	13,184 (35.2)	12,506 (33.6)	14,317 (37.3)	12,892 (36.8)	6,613 (32.1)	10,033 (36.9)	8,052 (35.3)	8,271 (36.7)
patients with T2DM with controlled HTN	23,903 (16.5)	20,004 (13.0)	20,649 (13.0)	19,323 (12.0)	11,889 (7.4)	15,014 (9.1)	15,851 (9.4)	17,581 (10.4)
Newly diagnosed diabetes complications [n (per 1000)]	650 (4.47)	1063 (6.92)	950 (5.96)	952 (5.93)	627 (3.92)	637 (3.86)	514 (3.05)	552 (3.25)
Refer to hospital emergency ward [n (per 1000]	1,569 (10.8)	1,408 (9.2)	1,651 (10.4)	1,518 (9.5)	976 (6.1)	1,333 (8.1)	1,176 (7.0)	1,491 (8.8)
Do not go to the health centers because of death [n (per 10,000)]	139 (9.6)	101 (6.6)	158 (9.9)	156 (9.7)	155 (9.7)	195 (11.8)	251 (14.9)	160 (9.4)
Do not go to the health centers because of other reasons [n (%)]	44,851 (30.9)	70,770 (46.0)	81,759 (51.3)	88,287 (55.0)	101,438 (63.4)	102,713 (62.2)	106,089 (62.9)	98,742 (58.2)

*T2DM* Type 2 diabetes mellitus, *COVID-19* Coronavirus disease 2019, *BMI* Body mass index, *FBS* Fasting blood sugar, *HbA1C* Hemoglobin glycated, *HTN* Hypertension

^a^ Number (percent)

**Fig. 1:**
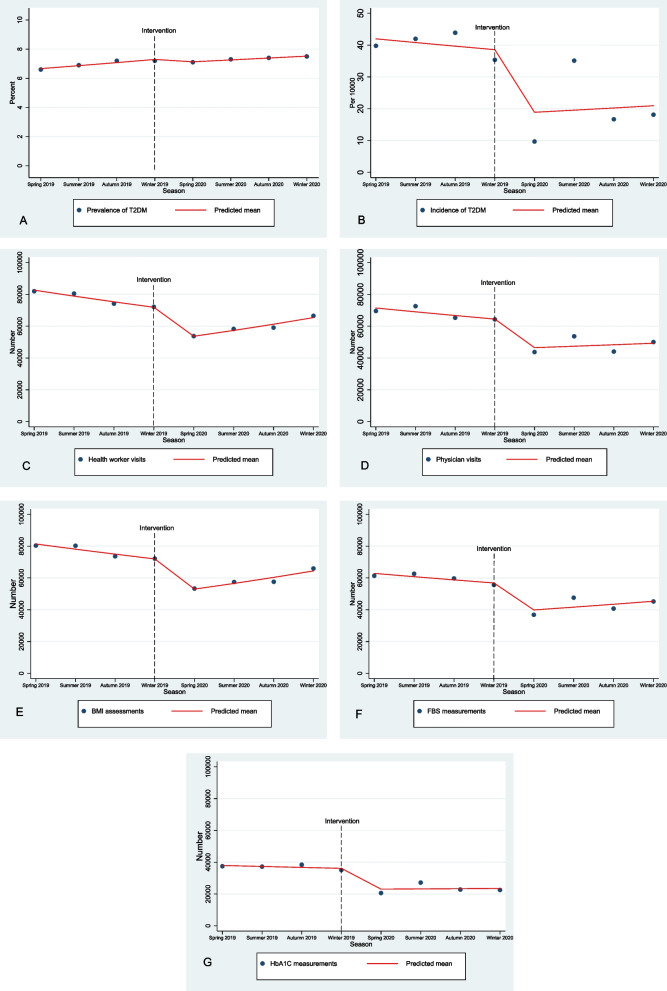
Interrupted Time Series analysis in health care indices in T2DM during 2019–2020 [**A** prevalence of T2DM, **B** incidence of T2DM, **C** health worker visits, **D** physician workers, **E** BMI assessments, **F** FBS measurements, **G** HbA1c measurements], Abbreviations: T2DM, Type 2 Diabetes Mellitus; BMI, Body Mass Index; FBS, Fasting Blood Sugar; HbA1C, Hemoglobin A1C

Table 3 shows trend, that induced by the COVID-19 pandemic, of diabetes care and outcomes. Both seasonal incidence (β1 = -0.028, *P* = 0.689; β2 = -0.685, *P* = 0.012; β3 = 0.062, *P* = 0.610) and prevalence (β1 = 0.030, *P* = 0.859; β2 = -0.052, *P* = 0.924; β3 = -0.012, *P* = 958) of T2DM was almost stable (not statistically changed) during the study period (Fig. 1 A, B).

**Table 3:** Interrupted Time Series analysis of number of T2DM care and health status indices during 2019–2020

Variable	Trend before COVID-19 pandemic		Level changed by COVID-19 natural intervention		Recovery (trend) during COVID-19 pandemic	
	β1 (95% CI)	P	β2 (95% CI)	P	β3 (95% CI)	P
Prevalence of T2DM [%]	0.030 (-0.30, 0.36)	0.859	-0.052 (-1.13, 1.03)	0.924	-0.012 (-0.47, 0.45)	0.958
Incidence of T2DM [Per 10000]	-.028 (-0.16, 0.10)	0.689	-0.685 (-1.22, -0.15)	0.012	0.062 (-0.17, 0.30)	0.610
Health worker visits [n]	-0.046 (-0.04, -0.04)	< 0.001	-0.245 (-0.25, -0.23)	< 0.001	0.112 (0.10, 0.11)	< 0.001
Physician visits [n]	-0.034 (-0.037, -0.030)	< 0.001	-0.292 (-0.30, -0.28)	< 0.001	0.053 (0.04, 0.05)	< 0.001
BMI assessment [n]	-0.04 (-0.04, -0.03)	< 0.001	-0.265 (-0.82, -0.76)	< 0.001	0.105 (0.10, 0.11)	< 0.001
FBS measurement [n]	-0.033 (-0.037, -0.030)	< 0.001	-0.320 (-0.33, -0.30)	< 0.001	0.076 (0.07, 0.08)	< 0.001
HbA1C measurement [n]	-0.016 (-0.02, -0.01)	< 0.001	-0.432 (-0.44, -0.41)	< 0.001	0.022 (0.01, 0.02)	< 0.001
HbA1C < 7% [%]	0.023 (-0.12, 0.17)	0.751	-0.125 (-0.61, 0.36)	0.614	0.010 (-0.19, 0.21)	0.919
patients with T2DM with controlled HTN [%]	-0.099 (-0.33, 0.13)	0.414	-0.312 (-1.20, 0.57)	0.492	0.202 (-0.17, 0.57)	0.294
Newly diagnosed diabetes complication [n]	0.087 (0.05, 0.11)	< 0.001	-0.566 (-0.66, -0.46)	< 0.001	-0.147 (-0.19, -0.10)	< 0.001
Refer to hospital emergency wards [n]	0.005 (-0.01, 0.02)	0.608	-0.401 (-0.48, -0.32)	< 0.001	0.106 (0.07, 0.13)	< 0.001
Do not go to the health centers because of death [n]	0.078 (0.003, 0.15)	0.040	0.069 (-0.16, 0.30)	0.559	-0.04 (-0.13, 0.05)	0.415
Do not go to the health centers because of other reasons [n]	0.200 (0.19, 0.20)	< 0.001	-0.109 (-0.11, -0.09)	< 0.001	-0.204 (-0.20, -0.20)	< 0.001

*T2DM* Type 2 diabetes mellitus, *COVID-19* Coronavirus disease 2019, *BMI* Body mass index, *FBS* Fasting blood sugar, *HbA1C* Hemoglobin glycated, *HTN* Hypertension

A significant decrease in the number of visits by health workers and physicians was observed before COVID-19 pandemic (β1 = -0.046, *P* < 0.001; β1 = -0.034, *P* < 0.001) which got worse by the first month of COVID-19 pandemic (β2 = -0.245, *P* < 0.001; β2 = -0.292, *P* < 0.001); but, it was recovered by following months (β3 = 0.112, *P* < 0.001; β3 = 0.053, *P* < 0.001) (Fig. 1C, D). The exact pattern was observed for number of BMI, FBS and HbA1c assessments (Table 3, Fig. 1 E- G).

A stable trend was observed for the proportion of T2DM patients with HbA1C < 7% (β1 = 0.023, *P* = 0.751; β2 = -0.125, *P* = 0.614; β3 = 0.010, *P* = 0.919) and controlled HTN during study period (β1 = -0.099, *P* = 0.414; β2 = -0.312, *P* = 0.492; β3 = 0.202, *P* = 0.294). But, a significant increasing pattern was observed in number of newly diagnosed diabetes complication before COVID-19 pandemic (β1 = 0.087, *P* < 0.001), but decreased after announcing the first cases of COVID-19 (β2 = -0.566, *P* < 0.001), and also by the next months (β3 = -0.147, *P* < 0.001) (Table 3).

Trend in number of refer to hospital emergency wards was stable and unchanged before the COVID-19 pandemic (β1 = 0.005, *P* = 0.608). By the emergence of COVID-19, this figure dropped significantly (β2 = -0.401, *P* < 0.001). Then, it increased in the next months (β3 = 0.106, *P* < 0.001) (Table 3). For interest of the readers, results of further analyses on the changes in diabetes care indices are yielded in two separate Supplementary files (file 1 contains 18 tables and file 2 contains 33 figures). Supplementary table 1 (table S1-S3) are description of population, epidemiological and diabetes care indices stratified by gender and season. The optimum and unsatisfactory health care indicators, divided by gender and season, are described in Table S4-6. Referring (refer to level 2 and hospital emergency ward) and not going to the health centers indices are described in Table S7-9 and are broken down by gender and season. Table S10-17 are Interrupted Time Series (ITS) analyses of just-mentioned indices based on the gender and season. Table S18 is the correlation of seasonal T2DM care numbers and seasonal rates of COVID-19 incidence rate, hospitalization and mortality rate during the first year of the COVID-19 pandemic in Fars province, Southern Iran. Supplementary Fig. 1 (figure S1-S33) are plots of ITS analyses on diabetes indices according to gender and season.

## Discussion

The healthcare service coverage is supposed to be affected by the outbreaks and epidemics, generally experiencing an early secular change (“Shock effect”) followed by a recovery trend toward pre-intervention levels (“System reaction”). The resources, social environment, and population behaviors all have a significant role in determining this recovery period's features, such as the recovery gap. According to reports, there is a declining trend in the coverage of diabetic healthcare services throughout the present COVID-19 pandemic [16–19]. Similarly, a significant decrease in the diabetes healthcare indices was found for number of visits by health workers and physicians, the number of BMI, FBS and HbA1c assessments, and number of refer to hospital emergency wards with the shock on the announcement of COVID-19 pandemic. Nonetheless, almost all of the measured indices started to significantly recover after a gap of one to two months. These changes in diabetes care visits and assessments might be associated with redistribution of health staff and facilities, lockdown policy, community fear and phobia (believes), and performance of mass media. The health surveillance system advised T2DM patients to self-monitor their blood sugar, live a healthy lifestyle, be instructed to drink enough water, exercise during lockdowns, use alternative strategies like telehealth, virtual and digital services, and have regular and continuous follow-up with efficient educational programs in order to improve the coverage of diabetes care [13].

Though only 4 times before the interruption is insufficient for trend analysis, a significant decreasing trend was present in number of visits by health workers and physicians, as well as BMI, FBS and HbA1C measurements before COVID-19 pandemic. One can emphasize on some weakness in our primary and secondary health system probably related to the weakness in the health economy, the disproportionate distribution of medical services and professionals, and health system bias toward tertiary care and. Regarding this situation, the COVID-19 pandemic's converging force might exacerbate this healthcare delivery deficiency. The patients with non-communicable illnesses, particularly those with T2DM, were more at risk when the majority of healthcare resources were diverted to the treatment of COVID-19 patients. Besides, many patients with such chronic conditions avoided their direct exposure to medical centers because of the risk of infection or the widespread fear and rumors. Furthermore, restrictions and quarantine measures established by the governments could disrupt self-care activities, insufficient clinical support and less monitoring of these patients [13, 23].

In theory, these changes would affect glycemic control and diabetes outlooks. In two reports from India and Singapore, no significant change in HbA1c and BMI mean levels before and after COVID-19 pandemic [24, 25]. These findings might be explained by the healthy lifestyles (healthy eating and exercise) and a brief observation period [26, 27]. In our study, the mean seasonal number of T2DM patients with controlled BMI, FBS, HbA1c, and blood pressure had decreased in 2020 compared to 2019 for each pair of similar seasons. However, these decreases may simply reflect a lower number of records in 2020 rather than an actual decrease in the number of patients with glycemic control following the COVID-19 pandemic. Hence, we showed that the proportions of HbA1C < 7% and controlled HTN did not significantly changed with the onset of the COVID-19 pandemic or in the following seven months. Noticeably, a portion of these secular trends should be attributed to the diabetes surveillance system purposely decisions. For example, during the pandemic, telecommunication and self-monitoring were suggested. We showed that the number of T2DM patients without any referral (excluding died patients) was statistically increased by starting COVID-19 pandemic; but the number of mortality-related do not go to the health centers and newly diagnosed diabetes complication were not statistically increased. Hence, it would be plausible to observe a secular trend in healthcare coverage without any significant change in outcomes. Although this statement should be considered with caution. For instance, it is well recognized [9, 28, 29] that mortality and mortality-related avoidance of health facilities is dramatically increasing. Or, Khader et al. [19] showed that the majority of T2DM patients who underwent home monitoring during COVID-19 epidemic had uncontrolled blood glucose levels. Besides, the mean incidence of diabetes was 19.91 per 10,000 people in 2020, which was lower than the mean of 40.27 per 10,000 people in 2019 (40.27 per 10 thousand). It might be related to the less disease screening and detection during COVID-19 pandemic, as well as the introduction of unusual nationwide policies (i.e., social distancing measures- Applying the limitations, implementing lockdowns, and remaining at home may interfere with the procedure for locating new patients. While the average prevalence of T2DM in 2019–2020 was 7.2%, our research team recently released a study in the province of Fars where we evaluated the prevalence of T2DM to be 7.2% and 8.74% in 2015 and 2016 [30].

The major strength is its’ large population (~ 160,000), while most of in-line studies are conducted on a substantially lower number of subjects. The major limitation was the lack of minimum time unit required for Interrupted Time Series analysis. Penfold and Zhang have stated that a minimum of eight events is required for before, and after the intervention to achieve robust result. Our data were inherently seasonal, which yielded four time-units for both before and after the intervention. Although interrupted time series analysis can be done with a smaller number of time units [31]. Furthermore, our data were inherently administrative – not for research purpose – that might be inevitably defective during the record process by health staff.

## Conclusions

We performed a large-scale ecologic study in southern Iran to assess the change in diabetes healthcare indices induced by the COVID-19 pandemic. In conclusion, we discovered that the mean incidence of diabetes decreased from 2019 to 2020. T2DM prevalence was 7.2% on average in 2019–2020.Besides, the healthcare coverage sharply declined with the announcement of COVID-19 pandemic, due to the shock effect. However, soon started to recover despite a gap of one to two months. Our data showed no significant changes in the diabetes outcomes, which might imply on the health surveillance system reaction. Finally, it is advised to adopt alternate tactics like telemedicine, digital services for consultations, self-management, remote monitoring, etc. to continue diabetes treatment while facing a pandemic status in the future. Besides, with a consistency in the health surveillance system, it would be more plausible to tackle issues confronting with unexpected massive challenges. Maintaining healthcare access for all should be a key priority in public health.

## Supplementary Information

**Additional file 1: Table S1.** population indices in patient with type 2 diabetes during 2019-2020. **Table S2.** Epidemiological indices in patient with T2DM during 2019-2020. **Table S3.** Frequency of performed diabetes care in patient with T2DM during 2019-2020. **Table S4.** Frequency of optimal and suboptimal BMI in type 2 diabetes during 2019-2020. **Table S5.** Frequency of optimal and suboptimal FBS in type 2 diabetes during 2019-2020. **Table S6.** Frequency of optimal and suboptimal HbA1C in type 2 diabetes during 2019-2020. **Table S7.** Frequency of Diabetic with HTN and HTN well control in type 2 diabetes during 2019-2020. **Table S8.** Frequency of new complications, refer to level 2 and emergency refer in type 2 diabetes during 2019-2020. **Table S9.** Frequency of do not go to the health centers due to death, migrate and other reasons in type 2 diabetes during 2019-2020. **Table S10.** Interrupted time series analysis for epidemiological indices in patient with T2DM during 2019-2020. **Table S11.** Interrupted time series analysis for performed diabetes care indices in patient with type 2 diabetes during 2019-2020. **Table S12.** Interrupted time series analysis in patient with type 2 diabetes by optimal and suboptimal BMI during 2019-2020. **Table S13.** Interrupted time series analysis in patient with type 2 diabetes by FBS during 2019-202. **Table S14.** Interrupted time series analysis in patient with type 2 diabetes by HbA1C during 2019-2020. **Table S15.** Interrupted time series analysis in patient with type 2 diabetes by HTN and HTN well control during 2019-2020. **Table S16.** Interrupted time series analysis in patient with type 2 diabetes by new Complication, refer to level 2 and emergency refer during 2019-2020. **Table S17.** Interrupted time series analysis in patient with type 2 diabetes by do not go to the health centers due to death, migrate and other reasons during 2019-2020. **Table S18.** Correlation of seasonal T2DM care numbers and seasonal rates of the COVID-19 incidence rate, hospitalization and mortality rate during the first year of the COVID-19 pandemic in Fars province, Southern Iran.

**Additional file 2:** **Figure S1.** Prevalence of T2DM in female during 2019-2020. **Figure S2.** Prevalence of T2DM in male during 2019-2020. **Figure S3.** Incidence rate of T2DM in female during 2019-2020. **Figure S4.** Incidence rate of T2DM in male during 2019-2020. **Figure S5.** Number of performed visit health worker in female during 2019-2020. **Figure S6.** Number of performed visit health worker in male during 2019-2020. **Figure S7.** Number of performed visit doctor in female during 2019-2020. **Figure S8.** Number of performed visit doctor in male during 2019-2020. **Figure S9.** Number of measured BMI in female during 2019-2020. **Figure S10.** Number of measured BMI in male during 2019-2020. **Figure S11.** Number of performed FBS in female during 2019-2020. **Figure S12.** Number of performed FBS in male during 2019-2020. **Figure S13.** Number of performed HbA1C in female during 2019-2020. **Figure S14.** Number of performed HbA1C in male during 2019-2020. **Figure S15.** Percent of BMI < 25 in patient with T2DM during 2019-2020. **Figure S16.** Percent of BMI 25-30 in patient with T2DM during 2019-2020. **Figure S17.** Percent of BMI ≥ 30 in patient with T2DM during 2019-2020. **Figure S18.** Percent of FBS < 70 in patient with T2DM during 2019-2020. **Figure S19.** Percent of FBS 70-130 in patient with T2DM during 2019-2020. **Figure S20.** Percent of FBS ≥ 130 in patient with T2DM during 2019-2020. **Figure S21.** Percent of HbA1C < 7 in patient with T2DM during 2019-2020. **Figure S22.** Percent of HbA1C 7-7.5 in patient with T2DM during 2019-2020. **Figure S23.** Percent of HbA1C 7.5-8 in patient with T2DM during 2019-2020. **Figure S24.** Percent of HbA1C 8-8.5 in patient with T2DM during 2019-2020. **Figure S25.** Percent of HbA1C ≥ 8.5 in patient with T2DM during 2019-2020. **Figure S26.** Number of diabetics with HTN in patient with T2DM during 2019-2020. **Figure S27.** Percent of diabetics with HTN well control in patient with T2DM during 2019-2020. **Figure S28.** Number of new complications of diabetes in patient with T2DM during 2019-2020. **Figure S29.** Number of refer to level 2 in patient with T2DM during 2019-2020. **Figure S30.** Number of emergency refer in patient with T2DM during 2019-2020. **Figure S31.** Number of do not go to the health centers due to death in patient with T2DM during 2019-2020. **Figure S32.** Number of do not go to the health centers due to migrate in patient with T2DM during 2019-2020. **Figure S33.** Number of do not go to the health centers due to other reasons in patient with T2DM during 2019-2020.

## Data Availability

The data that support this study’s findings are available from the Department of Noncommunicable disease Center at Shiraz University of Medical Science, but restrictions apply to the availability of these data, which were used under license for the current study, and so are not publicly available. But, data are available from corresponding author on reasonable request. Email address: afshinmohammadi295@yahoo.com.

## Abbreviations

T2DM: Type 2 diabetes mellitus
COVID-19: Coronavirus disease 2019
HTN: Hypertension
BP: Blood pressure
HbA1C: Hemoglobin A1c
BMI: Body mass index
FBS: Fasting blood sugar
CI: Confidence interval
ITS: Interrupted time series
