# Supplementary material for: The effect of COVID-19 pandemic on diabetes care indices in Southern Iran: an interrupted time series analysis
Source: BMC Health Serv Res. 2023 Feb 13;23:148. doi: 10.1186/s12913-023-09158-4 (PMC9925215; doi:10.1186/s12913-023-09158-4)
Supplement: Supplementary file 1 — Additional file 1: Table S1. population indices in patient with type 2 diabetes during 2019-2020. Table S2. Epidemiological indices in patient with T2DM during 2019-2020. Table S3. Frequency of performed diabetes care in patient with T2DM during 2019-2020. Table S4. Frequency of optimal and suboptimal BMI in type 2 diabetes during 2019-2020. Table S5. Frequency of optimal and suboptimal FBS in type 2 diabetes during 2019-2020. Table S6. Frequency of optimal and suboptimal HbA1C in type 2 diabetes during 2019-2020. Table S7. Frequency of Diabetic with HTN and HTN well control in type 2 diabetes during 2019-2020. Table S8. Frequency of new complications, refer to level 2 and emergency refer in type 2 diabetes during 2019-2020. Table S9. Frequency of do not go to the health centers due to death, migrate and other reasons in type 2 diabetes during 2019-2020. Table S10. Interrupted time series analysis for epidemiological indices in patient with T2DM during 2019-2020. Table S11. Interrupted time series analysis for performed diabetes care indices in patient with type 2 diabetes during 2019-2020. Table S12. Interrupted time series analysis in patient with type 2 diabetes by optimal and suboptimal BMI during 2019-2020. Table S13. Interrupted time series analysis in patient with type 2 diabetes by FBS during 2019-202. Table S14. Interrupted time series analysis in patient with type 2 diabetes by HbA1C during 2019-2020. Table S15. Interrupted time series analysis in patient with type 2 diabetes by HTN and HTN well control during 2019-2020. Table S16. Interrupted time series analysis in patient with type 2 diabetes by new Complication, refer to level 2 and emergency refer during 2019-2020. Table S17. Interrupted time series analysis in patient with type 2 diabetes by do not go to the health centers due to death, migrate and other reasons during 2019-2020. Table S18. Correlation of seasonal T2DM care numbers and seasonal rates of the COVID-19 incidence rate, hospitalization an [file 12913_2023_9158_MOESM1_ESM.docx]

**Table S1** population indices in patient with type 2 diabetes during 2019-2020

| **Year**  **Index** | | **2019** | | | | **2020** | | | |
| --- | --- | --- | --- | --- | --- | --- | --- | --- | --- |
|  |  | **Spring** | **Summer** | **Autumn** | **Winter** | **Spring** | **Summer** | **Autumn** | **Winter** |
| **Total population [n]** | **Female** | 2,039,733 | 2,085,864 | 2,088,745 | 2,089,043 | 2,077,130 | 2,128,471 | 2,128,443 | 2,128,443 |
|  | **Male** | 2,018,263 | 2,071,013 | 2,072,303 | 2,071,402 | 2,065,557 | 2,123,073 | 2,123,086 | 2,126,611 |
|  | **Total** | 4,057,996 | 4,156,877 | 4,161,048 | 4,160,445 | 4,142,687 | 4,251,544 | 4,251,529 | 4,255,054 |
|  | **Urban** | 2,956,990 | 3,034,509 | 3,040,175 | 3,039,280 | 3,016,843 | 3,098,098 | 3,098,255 | 3,101,780 |
|  | **Rural** | 1,101,006 | 1,122,368 | 1,120,873 | 1,121,165 | 1,125,844 | 1,153,446 | 1,153,274 | 1,153,274 |
| **Population ≥ 30 years [n]** | **Female** | 1,112,672 | 1,137,509 | 1,124,994 | 1,124,709 | 1,133,161 | 1,125,324 | 1,146,249 | 1,146,249 |
|  | **Male** | 1,084470 | 1,092,201 | 1,091,774 | 1,091,762 | 1,117,044 | 1,124,482 | 1,125,454 | 1,125,454 |
|  | **Total** | 2,197,142 | 2,229,710 | 2,216,768 | 2,216,471 | 2,250,205 | 2,249,806 | 2,271,703 | 2,271,703 |
|  | **Urban** | 1,640,504 | 1,672,772 | 1,663,569 | 1,663,834 | 1,689,420 | 1,698,649 | 1,719,653 | 1,719,653 |
|  | **Rural** | 556,638 | 556,938 | 553,199 | 552,637 | 560,785 | 551,157 | 552,050 | 552,050 |
| **T2DM population ≥ 30years [n]** | **Female** | 91,573 | 94,692 | 97,957 | 99,386 | 100,353 | 102,266 | 104,195 | 102,966 |
|  | **Male** | 53,709 | 58,992 | 61,373 | 61,078 | 59,739 | 62,932 | 64,593 | 66,659 |
|  | **Total** | 145,282 | 153,684 | 159,330 | 160,464 | 160,092 | 165,198 | 168,788 | 169,625 |
|  | **Urban** | 115,477 | 121,633 | 126,426 | 127,942 | 127,856 | 132,393 | 133,312 | 134,688 |
|  | **Rural** | 29,805 | 32,051 | 32,904 | 32,522 | 32,236 | 32,805 | 35,476 | 34,937 |
| **New T2DM population ≥ 30 [n]** | **Female** | 4,731 | 4,938 | 4,958 | 3,839 | 1,320 | 4,390 | 2,037 | 1,674 |
|  | **Male** | 3,469 | 3,815 | 4,115 | 3,461 | 709 | 2,958 | 1,478 | 2,143 |
|  | **Total** | 8,200 | 8,753 | 9,073 | 7,300 | 2,029 | 7,348 | 3,515 | 3,817 |
|  | **Urban** | 6,844 | 7,281 | 8,108 | 6,339 | 1,444 | 5,772 | 2,306 | 2,162 |
|  | **Rural** | 1,356 | 1,472 | 965 | 961 | 585 | 1,576 | 1,209 | 1,655 |

**Table S2** Epidemiological indices in patient with T2DM during 2019-2020

| **Year**  **Index** | | **2019** | | | | **2020** | | | |
| --- | --- | --- | --- | --- | --- | --- | --- | --- | --- |
|  |  | **Spring** | **Summer** | **Autumn** | **Winter** | **Spring** | **Summer** | **Autumn** | **Winter** |
| **Prevalence of T2DM in ≥ 30 years [%]** | **Female** | 8.2 | 8.3 | 8.7 | 8.8 | 8.9 | 9.1 | 9.1 | 9.0 |
|  | **Male** | 5.0 | 5.4 | 5.6 | 5.6 | 5.3 | 5.6 | 5.7 | 5.9 |
|  | **Total** | 6.6 | 6.9 | 7.2 | 7.2 | 7.1 | 7.3 | 7.4 | 7.5 |
|  | **Urban** | 7.0 | 7.3 | 7.6 | 7.7 | 7.6 | 7.8 | 7.8 | 7.8 |
|  | **Rural** | 5.4 | 5.8 | 5.9 | 5.9 | 5.7 | 6.0 | 6.4 | 6.3 |
| **Incidence of T2DM in ≥ 30 years [per 10000]** | **Female** | 46.1 | 47.1 | 48.1 | 37.3 | 12.8 | 42.8 | 19.5 | 16.1 |
|  | **Male** | 33.5 | 36.8 | 39.8 | 33.4 | 6.7 | 27.8 | 13.9 | 20.1 |
|  | **Total** | 39.8 | 41.9 | 43.9 | 35.4 | 9.7 | 35.1 | 16.7 | 18.1 |
|  | **Urban** | 44.7 | 46.7 | 52.4 | 41.1 | 9.2 | 36.7 | 14.5 | 13.6 |
|  | **Rural** | 25.7 | 27.9 | 18.5 | 18.4 | 11.1 | 30.3 | 23.3 | 31.9 |

**Table S3** Frequency of performed diabetes care in patient with T2DM during 2019-2020

| **Year**  **Index** | | **2019** | | | | **2020** | | | |
| --- | --- | --- | --- | --- | --- | --- | --- | --- | --- |
|  |  | **Spring** | **Summer** | **Autumn** | **Winter** | **Spring** | **Summer** | **Autumn** | **Winter** |
| **Performed visit health worker [n, %]** | **Female** | 52,298  57.1 | 51,923  54.8 | 47,783  48.8 | 45,830  46.1 | 35,245  35.1 | 37,693  36.9 | 38,120  36.6 | 41,813  40.6 |
|  | **Male** | 29,689  55.3 | 28,587  48.5 | 26,398  43.0 | 26,309  43.1 | 18,516  31.0 | 20,587  32.7 | 20,986  31.7 | 24,717  37.1 |
|  | **Total** | 81,987  56.4 | 80,510  52.4 | 74,181  46.6 | 72,139  45.0 | 53,761  33.6 | 58,280  35.3 | 59,106  35.0 | 66,530  39.2 |
|  | **Urban** | 55,018  47.6 | 53,217  43.8 | 50,373  39.8 | 49,457  38.7 | 32,907  25.7 | 37,240  28.1 | 35,459  26.6 | 39,331  29.2 |
|  | **Rural** | 26,969  90.5 | 27,293  85.2 | 23,808  72.4 | 22,682  69.7 | 20,854  64.7 | 21,040  64.1 | 23,647  66.7 | 27,199  77.9 |
| **Performed visit doctor [n, %]** | **Female** | 44,593  48.7 | 46,310  48.9 | 41,367  42.2 | 40,787  41.0 | 28,056  28.0 | 33,967  33.2 | 28,058  26.9 | 30,573  29.7 |
|  | **Male** | 24,973  46.5 | 26,260  44.5 | 23,927  39.0 | 23,479  38.4 | 15,711  26.3 | 19,691  31.3 | 16,004  24.8 | 19,476  29.2 |
|  | **Total** | 69,566  47.9 | 72,570  47.2 | 65,294  41.0 | 64,266  40.1 | 43,767  27.3 | 53,658  32.5 | 44,062  26.1 | 50,049  29.5 |
|  | **Urban** | 48,023  41.6 | 50,373  41.4 | 46,653  36.9 | 45,767  35.8 | 29,777  23.3 | 37,694  28.5 | 29,259  21.9 | 32,444  24.1 |
|  | **Rural** | 21,543  72.3 | 22,197  69.3 | 18,641  56.7 | 18,499  56.9 | 13,990  43.4 | 15,964  48.7 | 14,803  41.7 | 17,605  50.4 |
| **Performed BMI [n, %]** | **Female** | 51,123  55.8 | 51,982  54.9 | 47,460  48.4 | 45,865  46.1 | 34,915  34.8 | 37,078  36.3 | 37,739  36.2 | 41,482  39.7 |
|  | **Male** | 29,244  54.4 | 28,269  47.9 | 26,147  42.6 | 26,286  43.0 | 18,399  30.8 | 20,362  32.4 | 19,845  30.7 | 24,473  38.3 |
|  | **Total** | 80,367  55.3 | 80,251  52.2 | 73,607  46.2 | 72,151  45.0 | 53,314  33.3 | 57,440  34.8 | 57,584  34.1 | 65,955  39.2 |
|  | **Urban** | 54,719  47.4 | 53,004  43.6 | 50,039  39.6 | 49,562  38.7 | 32,790  25.6 | 37,100  28.0 | 35,057  26.3 | 39,288  29.2 |
|  | **Rural** | 25,648  86.1 | 27,247  85.0 | 23,568  71.6 | 22,589  69.5 | 20,524  63.7 | 20,340  62.0 | 22,527  63.5 | 26,667  76.3 |
| **Performed FBS [n, %]** | **Female** | 38,599  42.2 | 39,784  42.0 | 37,302  38.1 | 34,998  35.2 | 23,383  23.3 | 29,939  29.3 | 25,881  24.8 | 28,046  26.9 |
|  | **Male** | 22,745  42.3 | 22,834  38.7 | 22,400  36.5 | 20,621  33.8 | 13,463  22.5 | 17,628  28.0 | 14,844  23.0 | 17,149  26.8 |
|  | **Total** | 61,344  42.2 | 62,618  40.7 | 59,702  37.5 | 55,619  34.7 | 36,846  23.0 | 47,567  28.8 | 40,725  24.1 | 45,195  26.8 |
|  | **Urban** | 44,448  38.5 | 45,245  37.2 | 44,518  35.2 | 41,294  32.3 | 25,420  19.9 | 35,089  26.5 | 27,789  20.8 | 31,009  23.0 |
|  | **Rural** | 16,896  56.7 | 17,373  54.2 | 15,184  46.1 | 14,325  44.0 | 11,426  35.4 | 12,478  38.0 | 12,936  36.5 | 14,186  40.6 |
| **Performed HbA1C [n, %]** | **Female** | 22,682  24.8 | 23,322  24.6 | 23,440  23.9 | 21,101  21.2 | 12,919  12.9 | 16,346  16.0 | 13,812  13.3 | 12,430  11.9 |
|  | **Male** | 14,776  27.5 | 13,862  23.5 | 14,968  24.4 | 13,942  22.8 | 7,682  12.9 | 10,816  17.2 | 8,973  13.9 | 10,078  15.8 |
|  | **Total** | 37,458  25.8 | 37,184  24.2 | 38,408  24.1 | 35,043  21.8 | 20,601  12.9 | 27,162  16.4 | 22,785  13.5 | 22,508  13.4 |
|  | **Urban** | 30,163  26.1 | 30,027  24.7 | 31,277  24.7 | 27,994  21.9 | 15,365  12.0 | 21,332  16.1 | 17,309  13.0 | 16,379  12.2 |
|  | **Rural** | 7,295  24.5 | 7,157  22.3 | 7,131  21.7 | 7,049  21.7 | 5,236  16.2 | 5,830  17.8 | 5,476  15.4 | 6,129  17.5 |
| ***N**  **%** | | | | | | | | | |

**Table S4** Frequency of optimal and suboptimal BMI in type 2 diabetes during 2019-2020

| **Year**  **Index** | | **2019** | | | | **2020** | | | |
| --- | --- | --- | --- | --- | --- | --- | --- | --- | --- |
|  |  | **Spring** | **Summer** | **Autumn** | **Winter** | **Spring** | **Summer** | **Autumn** | **Winter** |
| **BMI < 25  [n, %]** | **Female** | 13,114  25.7 | 14,217  27.3 | 13,280  28.0 | 12,832  28.0 | 10,230  29.3 | 11,103  29.9 | 10,427  27.6 | 12,091  29.1 |
|  | **Male** | 8,156  27.9 | 8,671  30.7 | 8,303  31.8 | 8,083  30.8 | 5,983  32.5 | 6,420  31.5 | 6,170  31.1 | 7,321  29.9 |
|  | **Total** | 21,270  26.5 | 22,888  28.5 | 21,583  29.3 | 20,915  29.0 | 16,213  30.4 | 17,523  30.5 | 16,597  28.8 | 19,412  29.4 |
|  | **Urban** | 13,240  24.2 | 14,251  26.9 | 13,871  27.7 | 13,505  27.2 | 9,643  29.4 | 11,126  30.0 | 9,820  28.0 | 11,248  28.6 |
|  | **Rural** | 8,030  31.3 | 8,637  31.7 | 7,712  32.7 | 7,410  32.8 | 6,570  32.0 | 6,397  31.5 | 6,777  30.1 | 8,164  30.6 |
| **BMI 25-30  [n, %]** | **Female** | 25,989  50.8 | 25,240  48.6 | 23,321  49.1 | 22,737  49.6 | 16,823  48.2 | 17,388  46.9 | 19,267  51.1 | 20,319  49.0 |
|  | **Male** | 14,714  50.3 | 13,480  47.7 | 12,461  47.7 | 12,760  48.5 | 8,744  47.5 | 9,905  48.6 | 9,613  48.4 | 12,296  50.2 |
|  | **Total** | 40,703  50.6 | 38,720  48.2 | 35,782  48.6 | 35,497  49.2 | 25,567  48.0 | 27,293  47.5 | 28,880  50.2 | 32,615  49.5 |
|  | **Urban** | 28,680  52.4 | 26,254  49.5 | 24,811  49.6 | 24,695  49.8 | 15,517  47.3 | 17,661  47.6 | 17,808  50.8 | 19,759  50.3 |
|  | **Rural** | 12,023  46.9 | 12,466  45.8 | 10,971  46.6 | 10,802  47.8 | 10,050  49.0 | 9,632  47.4 | 11,072  49.1 | 12,856  48.2 |
| **BMI ≥ 30  [n, %]** | **Female** | 12,020  23.5 | 12,525  24.1 | 10,859  22.9 | 10,296  22.4 | 7,862  22.5 | 8,587  23.2 | 8,045  21.3 | 9,072  21.9 |
|  | **Male** | 6,374  21.8 | 6,118  21.6 | 5,383  20.6 | 5,443  20.7 | 3,672  20.0 | 4,037  19.8 | 4,062  20.5 | 4,856  19.8 |
|  | **Total** | 18,394  22.9 | 18,643  23.2 | 16,242  22.1 | 15,739  21.8 | 11,534  21.6 | 12,624  22.0 | 12,107  21.0 | 13,928  21.1 |
|  | **Urban** | 12,799  23.4 | 12,499  23.6 | 11,357  22.7 | 11,362  22.9 | 7,630  23.3 | 8,313  22.4 | 7,429  21.2 | 8,281  21.1 |
|  | **Rural** | 5,595  21.8 | 6,144  22.5 | 4,885  20.7 | 4,377  19.4 | 3,904  19.0 | 4,311  21.2 | 4,678  20.8 | 5,647  21.2 |

**Table S5** Frequency of optimal and suboptimal FBS in type 2 diabetes during 2019-2020

| **Year**  **Index** | | **2019** | | | | **2020** | | | |
| --- | --- | --- | --- | --- | --- | --- | --- | --- | --- |
|  |  | **Spring** | **Summer** | **Autumn** | **Winter** | **Spring** | **Summer** | **Autumn** | **Winter** |
| **FBS < 70  [n, %]** | **Female** | 3,886  10.1 | 4,474  11.2 | 3,904  10.5 | 3,903  11.2 | 2,826  12.1 | 4,391  14.7 | 3,050  11.8 | 3,097  11.0 |
|  | **Male** | 2,733  12.0 | 3,253  14.2 | 2,743  12.2 | 2,627  12.7 | 1,633  12.1 | 2,442  13.9 | 1,841  12.4 | 2,191  12.8 |
|  | **Total** | 6,619  10.8 | 7,727  12.3 | 6,647  11.1 | 6,530  11.7 | 4,459  12.1 | 6,833  14.4 | 4,891  12.0 | 5,288  11.7 |
|  | **Urban** | 4,684  10.5 | 5,893  13.0 | 5,537  12.4 | 5,546  13.4 | 3,518  13.8 | 5,525  15.7 | 3,728  13.4 | 3,950  12.7 |
|  | **Rural** | 1,935  11.5 | 1,834  10.6 | 1,110  7.3 | 984  6.9 | 941  8.2 | 1,308  10.5 | 1,163  9.0 | 1,338  9.4 |
| **FBS 70-130 [n, %]** | **Female** | 21,825  56.5 | 21,714  54.6 | 21,086  56.5 | 20,313  58.0 | 12,911  55.2 | 16,534  55.2 | 15,183  58.7 | 16,020  57.1 |
|  | **Male** | 13,095  57.6 | 12,031  52.7 | 12,481  55.7 | 11,869  57.6 | 7,222  53.6 | 10,099  57.3 | 8,540  57.5 | 9,921  57.9 |
|  | **Total** | 34,920  56.9 | 33,745  53.9 | 33,567  56.2 | 32,182  57.9 | 20,133  54.6 | 26,633  56.0 | 23,723  58.3 | 25,941  57.4 |
|  | **Urban** | 26,247  59.1 | 24,717  54.6 | 25,173  56.5 | 24,513  59.4 | 14,121  55.6 | 20,077  57.2 | 16,497  59.4 | 18,090  58.3 |
|  | **Rural** | 8,673  51.3 | 9,028  52.0 | 8,394  55.3 | 7,669  53.5 | 6,012  52.6 | 6,556  52.5 | 7,226  55.9 | 7,851  55.3 |
| **FBS ≥ 130  [n, %]** | **Female** | 12,888  33.4 | 13,596  34.2 | 12,312  33.0 | 10,782  30.8 | 7,646  32.7 | 9,014  30.1 | 7,648  29.6 | 8,929  31.8 |
|  | **Male** | 6,917  30.4 | 7,550  33.1 | 7,176  32.0 | 6,125  29.7 | 4,608  34.2 | 5,087  28.9 | 4,463  30.1 | 5,037  29.4 |
|  | **Total** | 19,805  32.3 | 21,146  33.8 | 19,488  32.6 | 16,907  30.4 | 12,254  33.3 | 14,101  29.6 | 12,111  29.7 | 13,966  30.9 |
|  | **Urban** | 13,517  30.4 | 14,635  32.3 | 13,808  31.0 | 11,235  27.2 | 7,781  30.6 | 9,487  27.0 | 7,564  27.2 | 8,969  28.9 |
|  | **Rural** | 6,288  37.2 | 6,511  37.5 | 5,680  37.4 | 5,672  39.6 | 4,473  39.1 | 4,614  37.0 | 4,547  35.1 | 4,997  35.2 |

**Table S6** Frequency of optimal and suboptimal HbA1C in type 2 diabetes during 2019-2020

| **Year**  **Index** | | **2019** | | | | **2020** | | | |
| --- | --- | --- | --- | --- | --- | --- | --- | --- | --- |
|  |  | **Spring** | **Summer** | **Autumn** | **Winter** | **Spring** | **Summer** | **Autumn** | **Winter** |
| **HbA1C < 7  [n, %]** | **Female** | 7,962  35.1 | 7,917  33.9 | 8,851  37.8 | 7,960  37.7 | 4,086  31.6 | 6,116  37.4 | 4,792  34.7 | 4,712  37.9 |
|  | **Male** | 5,222  35.3 | 4,589  33.1 | 5,466  36.5 | 4,932  35.4 | 2,527  32.9 | 3,917  36.2 | 3,260  36.3 | 3,559  35.3 |
|  | **Total** | 13,184  35.2 | 12,506  33.6 | 14,317  37.3 | 12,892  36.8 | 6,613  32.1 | 10,033  36.9 | 8,052  35.3 | 8,271  36.7 |
|  | **Urban** | 10,403  34.5 | 10,095  33.6 | 11,748  37.6 | 10,539  37.6 | 4,887  31.8 | 7,948  37.3 | 6,321  36.5 | 6,196  37.8 |
|  | **Rural** | 2,781  38.1 | 2,411  33.7 | 2,569  36.0 | 2,353  33.4 | 1,726  33.0 | 2,085  35.8 | 1,731  31.6 | 2,075  33.9 |
| **HbA1C 7-7.5  [n, %]** | **Female** | 6,583  29.0 | 6,475  27.8 | 6,727  28.7 | 6,191  29.3 | 4,562  35.3 | 4,985  30.5 | 4,336  31.4 | 3,818  30.7 |
|  | **Male** | 4,346  29.4 | 4,125  29.8 | 4,414  29.5 | 4,665  33.5 | 2,651  34.5 | 3,449  31.9 | 2,831  31.6 | 3,161  31.4 |
|  | **Total** | 10,929  29.2 | 10,600  28.5 | 11,141  29.0 | 10,856  31.0 | 7,213  35.0 | 8,434  31.1 | 7,167  31.5 | 6,979  31.0 |
|  | **Urban** | 8,922  29.6 | 8,551  28.5 | 9,135  29.2 | 8,869  31.7 | 5,778  37.6 | 6,646  31.2 | 5,347  30.9 | 5,294  32.3 |
|  | **Rural** | 2,007  27.5 | 2,049  28.6 | 2,006  28.1 | 1,987  28.2 | 1,435  27.4 | 1,788  30.7 | 1,820  33.2 | 1,685  27.5 |
| **HbA1C 7.5-8  [n, %]** | **Female** | 4,291  18.9 | 5,025  21.5 | 4,357  18.6 | 3,804  18.0 | 2,328  18.0 | 2,784  17.0 | 2,502  18.1 | 2,147  17.3 |
|  | **Male** | 2,836  19.2 | 2,568  18.5 | 2,731  18.2 | 2,391  17.1 | 1,350  17.6 | 1,888  17.5 | 1,538  17.1 | 1,783  17.7 |
|  | **Total** | 7,127  19.0 | 7,593  20.4 | 7,088  18.5 | 6,195  17.7 | 3,678  17.9 | 4,672  17.2 | 4,040  17.7 | 3,930  17.5 |
|  | **Urban** | 5,834  19.3 | 6,277  20.9 | 5,824  18.6 | 4,981  17.8 | 2,707  17.6 | 3,657  17.1 | 3,035  17.5 | 2,759  16.8 |
|  | **Rural** | 1,293  17.7 | 1,316  18.4 | 1,264  17.7 | 1,214  17.2 | 971  18.5 | 1,015  17.4 | 1,005  18.4 | 1,171  19.1 |
| **HbA1C 8-8.5  [n, %]** | **Female** | 2,177  9.6 | 2,261  9.7 | 2,060  8.8 | 2,008  9.5 | 1,114  8.6 | 1,358  8.3 | 1,263  9.1 | 1,103  8.9 |
|  | **Male** | 1,342  9.1 | 1,584  11.4 | 1,380  9.2 | 1,134  8.1 | 717  9.3 | 861  8.0 | 815  9.1 | 904  9.0 |
|  | **Total** | 3,519  9.4 | 3,845  10.3 | 3,440  9.0 | 3,142  9.0 | 1,831  8.9 | 2,219  8.2 | 2,078  9.1 | 2,007  8.9 |
|  | **Urban** | 2,849  9.4 | 3,133  10.4 | 2,715  8.7 | 2,195  7.8 | 1,264  8.2 | 1,702  8.0 | 1,526  8.8 | 1,271  7.8 |
|  | **Rural** | 670  9.2 | 712  9.9 | 725  10.2 | 947  13.4 | 567  10.8 | 517  8.9 | 552  10.1 | 736  12.0 |
| **HbA1C ≥ 8.5  [n, %]** | **Female** | 1,669  7.4 | 1,644  7.0 | 1,445  6.2 | 1,138  5.4 | 829  6.4 | 1,103  6.7 | 919  6.7 | 650  5.2 |
|  | **Male** | 1,030  7.0 | 996  7.2 | 977  6.5 | 820  5.9 | 437  5.7 | 701  6.5 | 529  5.9 | 671  6.7 |
|  | **Total** | 2,699  7.2 | 2,640  7.1 | 2,422  6.3 | 1,958  5.6 | 1,266  6.1 | 1,804  6.6 | 1,448  6.4 | 1,321  5.9 |
|  | **Urban** | 37,458  7.1 | 37,184  6.6 | 38,408  5.9 | 35,043  5.0 | 20,601  4.7 | 27,162  6.5 | 22,785  6.2 | 22,508  5.2 |
|  | **Rural** | 30,163  7.5 | 30,027  9.3 | 31,277  8.0 | 27,994  7.8 | 15,365  10.3 | 21,332  7.3 | 17,309  6.7 | 16,379  7.5 |

**Table S7** Frequency of Diabetic with HTN and HTN well control in type 2 diabetes during 2019-2020

| **Year**  **Index** | | **2019** | | | | **2020** | | | |
| --- | --- | --- | --- | --- | --- | --- | --- | --- | --- |
|  |  | **Spring** | **Summer** | **Autumn** | **Winter** | **Spring** | **Summer** | **Autumn** | **Winter** |
| **Diabetic with HTN [n, %]** | **Female** | 16,032  17.5 | 17,514  18.5 | 16,212  16.6 | 15,255  15.3 | 9,828  9.8 | 11,098  10.9 | 10,330  9.9 | 11,889  11.5 |
|  | **Male** | 10,238  19.1 | 10,040  17.0 | 9,993  16.3 | 9,202  15.1 | 6,164  10.3 | 6,602  10.5 | 5,720  8.9 | 7,576  11.4 |
|  | **Total** | 26,270  18.1 | 27,554  17.9 | 26,205  16.4 | 24,457  15.2 | 15,992  10.0 | 17,700  10.7 | 16,050  9.5 | 19,465  11.5 |
|  | **Urban** | 18,490  16.0 | 18,745  15.4 | 17,377  13.7 | 16,590  13.0 | 9,656  7.6 | 11,393  8.6 | 10,143  7.6 | 12,078  9.0 |
|  | **Rural** | 7,780  26.1 | 8,809  27.5 | 8,828  26.8 | 7,867  24.2 | 6,336  19.7 | 6,307  19.2 | 5,907  16.7 | 7,387  21.1 |
| **Diabetic with HTN well control [n, %]** | **Female** | 15,262  16.7 | 12,782  13.5 | 12,951  13.2 | 12,197  12.3 | 7,269  7.2 | 9,536  9.3 | 9,794  9.4 | 10,937  10.5 |
|  | **Male** | 8,641  16.1 | 7,222  12.2 | 7,698  12.5 | 7,126  11.7 | 4,620  7.7 | 5,478  8.7 | 6,057  9.4 | 6,644  10.4 |
|  | **Total** | 23,903  16.5 | 20,004  13.0 | 20,649  13.0 | 19,323  12.0 | 11,889  7.4 | 15,014  9.1 | 15,851  9.4 | 17,581  10.4 |
|  | **Urban** | 16,095  13.9 | 12,798  10.5 | 14,229  11.3 | 13,321  10.4 | 7,251  5.7 | 9,216  7.0 | 9,800  7.4 | 10,549  7.8 |
|  | **Rural** | 7,808  26.2 | 7,206  22.5 | 6,420  19.5 | 6,002  18.5 | 4,638  14.4 | 5,798  17.7 | 6,051  17.1 | 7,032  20.1 |

**Table S8** Frequency of new complications, refer to level 2 and emergency refer in type 2 diabetes during 2019-2020

| **Year**  **Index** | | **2019** | | | | **2020** | | | |
| --- | --- | --- | --- | --- | --- | --- | --- | --- | --- |
|  |  | **Spring** | **Summer** | **Autumn** | **Winter** | **Spring** | **Summer** | **Autumn** | **Winter** |
| **New complications  [n, %]** | **Female** | 395  4.31 | 612  6.46 | 514  5.25 | 533  5.36 | 344  3.43 | 365  3.57 | 256  2.46 | 322  3.13 |
|  | **Male** | 255  4.75 | 451  7.65 | 436  7.10 | 419  6.86 | 283  4.74 | 272  4.32 | 258  3.99 | 230  3.45 |
|  | **Total** | 650  4.47 | 1063  6.92 | 950  5.96 | 952  5.93 | 627  3.92 | 637  3.86 | 514  3.05 | 552  3.25 |
|  | **Urban** | 496  4.30 | 931  7.65 | 767  6.07 | 793  6.20 | 463  3.62 | 489  3.69 | 387  2.90 | 311  2.31 |
|  | **Rural** | 154  5.17 | 132  4.12 | 183  5.56 | 159  4.89 | 164  5.09 | 148  4.51 | 127  3.58 | 241  6.90 |
| **Refer to level 2 [n, %]** | **Female** | 3,221  3.5 | 5,822  6.1 | 8,151  8.3 | 7,487  7.5 | 3,594  3.6 | 5,270  5.2 | 5,307  5.1 | 5,964  5.7 |
|  | **Male** | 2,051  3.8 | 3,938  6.7 | 5,415  8.8 | 5,027  8.2 | 2,664  4.5 | 3,539  5.6 | 3,832  5.9 | 4,148  6.5 |
|  | **Total** | 5,272  3.6 | 9,760  6.4 | 13,566  8.5 | 12,514  7.8 | 6,258  3.9 | 8,809  5.3 | 9,139  5.4 | 10,112  6.0 |
|  | **Urban** | 3,330  2.9 | 7,159  5.9 | 11,138  8.8 | 10,215  8.0 | 4,873  3.8 | 6,721  5.1 | 7,175  5.4 | 8,038  6.0 |
|  | **Rural** | 1,942  6.5 | 2,601  8.1 | 2,428  7.4 | 2,299  7.1 | 1,385  4.3 | 2,088  6.4 | 1,964  5.5 | 2,074  5.9 |
| **Emergency refer [n, Per 1000]** | **Female** | 846  9.24 | 770  8.13 | 996  10.17 | 931  9.37 | 446  4.44 | 658  6.43 | 573  5.50 | 910  8.71 |
|  | **Male** | 723  13.46 | 638  10.82 | 655  10.67 | 587  9.61 | 530  8.87 | 675  10.73 | 603  9.34 | 581  9.09 |
|  | **Total** | 1,569  10.8 | 1,408  9.2 | 1,651  10.4 | 1,518  9.5 | 976  6.1 | 1,333  8.1 | 1,176  7.0 | 1,491  8.8 |
|  | **Urban** | 1,217  10.5 | 1,039  8.5 | 1,440  11.4 | 1,333  10.4 | 799  6.2 | 1,039  7.8 | 975  7.3 | 1,132  8.4 |
|  | **Rural** | 352  11.8 | 369  11.5 | 211  6.4 | 185  5.7 | 177  5.5 | 294  9.0 | 201  5.7 | 359  10.3 |

**Table S9** Frequency of do not go to the health centers due to death, migrate and other reasons in type 2 diabetes during 2019-2020

| **Year**  **Index** | | **2019** | | | | **2020** | | | |
| --- | --- | --- | --- | --- | --- | --- | --- | --- | --- |
|  |  | **Spring** | **Summer** | **Autumn** | **Winter** | **Spring** | **Summer** | **Autumn** | **Winter** |
| **Do not go to the health centers due to death  [n, Per 10000]** | **Female** | 68  7.4 | 58  6.1 | 89  9.1 | 90  9.1 | 78  7.8 | 114  11.1 | 127  12.2 | 87  8.4 |
|  | **Male** | 71  13.2 | 43  7.3 | 69  11.2 | 66  10.8 | 77  12.9 | 81  12.9 | 124  19.2 | 73  11.4 |
|  | **Total** | 139  9.6 | 101  6.6 | 158  9.9 | 156  9.7 | 155  9.7 | 195  11.8 | 251  14.9 | 160  9.4 |
|  | **Urban** | 79  6.8 | 53  4.4 | 91  7.2 | 99  7.7 | 82  6.4 | 113  8.5 | 120  9.0 | 97  7.2 |
|  | **Rural** | 60  20.1 | 48  15.0 | 67  20.4 | 57  17.5 | 73  22.6 | 82  25.0 | 131  36.9 | 62  17.7 |
| **Do not go to the health centers due to migrate  [n, Per 10000]** | **Female** | 106  11.6 | 269  28.4 | 195  19.9 | 229  23.0 | 199  19.8 | 189  18.5 | 172  16.5 | 203  19.7 |
|  | **Male** | 75  14.0 | 204  34.6 | 112  18.2 | 115  18.8 | 179  30.0 | 164  26.1 | 132  20.4 | 157  23.5 |
|  | **Total** | 181  12.5 | 473  30.8 | 307  19.3 | 344  21.4 | 378  23.6 | 353  21.4 | 304  18.0 | 360  21.2 |
|  | **Urban** | 123  10.7 | 348  28.6 | 215  17.0 | 272  21.3 | 287  22.4 | 249  18.8 | 218  16.4 | 307  22.8 |
|  | **Rural** | 58  19.5 | 125  39.0 | 92  28.0 | 72  22.1 | 91  28.2 | 104  31.7 | 86  24.2 | 53  15.2 |
| **Do not go to the health centers due to other reasons [n, %]** | **Female** | 26,870  29.3 | 41,499  43.8 | 48,145  49.1 | 52,336  52.7 | 61,737  61.5 | 61,754  60.4 | 64,226  61.6 | 57,578  55.9 |
|  | **Male** | 17,981  33.5 | 29,271  49.6 | 33,614  54.8 | 35,951  58.9 | 39,701  66.5 | 40,959  65.1 | 41,863  64.8 | 41,164  61.7 |
|  | **Total** | 44,851  30.9 | 70,770  46.0 | 81,759  51.3 | 88,287  55.0 | 101,438  63.4 | 102,713  62.2 | 106,089  62.9 | 98,742  58.2 |
|  | **Urban** | 41,691  36.1 | 67,136  55.2 | 73,812  58.4 | 79,846  62.4 | 91,406  71.5 | 93,286  70.5 | 95,950  72.0 | 91,078  67.6 |
|  | **Rural** | 3,160  10.6 | 3,634  11.3 | 7,947  24.2 | 8,441  26.0 | 10,032  31.1 | 9,427  28.7 | 10,139  28.6 | 7,664  21.9 |

**Table S10** Interrupted time series analysis for epidemiological indices in patient with T2DM during 2019-2020

| **Variable** | | **Trend before COVID-19 pandemic** | | **Level changed by COVID-19 natural intervention** | | **Recovery (trend) during COVID-19 pandemic** | |
| --- | --- | --- | --- | --- | --- | --- | --- |
|  |  | **β1**  **(95% CI)** | **p-value** | **β2**  **(95% CI)** | **p-value** | **β3**  **(95% CI)** | **p-value** |
| **Prevalence of T2DM (%)** | **Female** | .025  (-.27, .32) | 0.866 | -.009  (-0.99, 0.97) | 0.985 | -.022  (-.44, .39) | 0.916 |
|  | **Male** | .037  (-.34, .41) | 0.847 | -.102  (-1.33, 1.13) | 0.871 | -.003  (-.53, .52) | 0.99 |
|  | **Total** | .030  (-.30, .36) | 0.859 | -.052  (-1.13, 1.03) | 0.924 | -.012  (-.47, .45) | 0.958 |
|  | **Urban** | .032  (-.28, .35) | 0.844 | -.045  (-1.09, 1.006) | 0.932 | -.024  (-.47, .42) | 0.914 |
|  | **Rural** | .027  (-.33, .39) | 0.881 | -.064  (-1.26, 1.13) | 0.915 | .008  (-.50, .51) | 0.975 |
| **Incidence of T2DM  (Per 10000)** | **Female** | -.057  (-.18, .07) | 0.393 | -.442  (-0.93, 0.05) | 0.081 | -.001  (-.22, .22) | 0.987 |
|  | **Male** | .007  (-.13, .15) | 0.917 | -1.006  (-1.59, -0.41) | 0.001 | .147  (-.11, .40) | 0.264 |
|  | **Total** | -.028  (-.16, .10) | 0.689 | -0.685  (-1.22, -0.15) | 0.012 | .062  (-.17, .30) | 0.610 |
|  | **Urban** | -.010  (-.13, .11) | 0.870 | -.815  (-1.32, -.30) | 0.002 | -.038  (-.27, .20) | 0.757 |
|  | **Rural** | -.138  (-.32, .04) | 0.144 | 0.037  (-0.62, 0.69) | 0.910 | .371  (.11, .63) | 0.005 |

**Table S11** Interrupted time series analysis for performed diabetes care indices in patient with type 2 diabetes during 2019-2020

| **Variable** | | **Trend before COVID-19 pandemic** | | **Level changed by COVID-19 natural intervention** | | **Recovery (trend) during COVID-19 pandemic** | |
| --- | --- | --- | --- | --- | --- | --- | --- |
|  |  | **β1**  **(95% CI)** | **p-value** | **β2**  **(95% CI)** | **p-value** | **β3**  **(95% CI)** | **p-value** |
| **performed Visit health worker [n]** | **Female** | -0.047  (-0.05, -0.04) | < 0.001 | -0.218  (-0.23, -0.20) | < 0.001 | 0.100  (0.09, 0.10) | < 0.001 |
|  | **Male** | -0.044  (-0.04, -0.03) | < 0.001 | -0.296  (-0.31, -0.27) | < 0.001 | 0.134  (0.12, 0.14) | < 0.001 |
|  | **Total** | -0.046  (-0.20, 0.04) | < 0.001 | -0.245  (-0.25, -0.23) | < 0.001 | 0.112  (-0.06, 0.31) | < 0.001 |
|  | **Urban** | -.037  (-.04, -.03) | < 0.001 | -0.340  (-0.35, -0.32) | < 0.001 | .085  (.07, .09) | < 0.001 |
|  | **Rural** | -.064  (-.07, -.05) | < 0.001 | -0.063  (-0.08, -0.04) | < 0.001 | .158  (.15, .16) | < 0.001 |
| **performed Visit doctor [n]** | **Female** | -0.037  (-.04, -0.03) | < 0.001 | -0.273  (-0.28, -0.25) | < 0.001 | 0.043  (.03, 0.04) | < 0.001 |
|  | **Male** | -0.027  (-0.03, -0.02) | < 0.001 | -0.326  (-0.34, -0.30) | < 0.001 | 0.070  (0.06, 0.07) | < 0.001 |
|  | **Total** | -0.034  (-0.19, 0.06) | < 0.001 | -0.292  (-0.30, -0.28) | < 0.001 | 0.053  (-0.14, 0.27) | < 0.001 |
|  | **Urban** | -.021  (-.02, -.01) | < 0.001 | -0.332  (-0.34, -0.31) | < 0.001 | .020  (.01, .02) | < 0.001 |
|  | **Rural** | -.062  (-.06, -.05) | < 0.001 | -0.196  (-0.21, -0.17) | < 0.001 | .125  (.11, .13) | < 0.001 |
| **performed BMI [n]** | **Female** | -0.041  (-0.04, -0.03) | < 0.001 | -0.239  (-0.25, -0.22) | < 0.001 | 0.095  (0.08, 0.10) | < 0.001 |
|  | **Male** | -0.040  (-0.04, -0.03) | < 0.001 | -0.311  (-0.33, -0.29) | < 0.001 | 0.125  (0.11, 0.13) | < 0.001 |
|  | **Total** | -0.040  (-0.19, 0.05) | < 0.001 | -0.265  (-0.27, -0.25) | < 0.001 | 0.105  (-0.07, 0.31) | < 0.001 |
|  | **Urban** | -.035  (-.03, -.03) | < 0.001 | -0.347  (-0.36, -0.33) | < 0.001 | .084  (.07, .09) | < 0.001 |
|  | **Rural** | -.051  (-.05, -.04) | < 0.001 | -0.106  (-0.12, -0.08) | < 0.001 | .143  (.13, .15) | < 0.001 |
| **performed FBS [n]** | **Female** | -0.035  (-0.03, -0.03) | < 0.001 | -0.307  (-0.32, -0.29) | < 0.001 | .072  (0.06, 0.07) | < 0.001 |
|  | **Male** | -0.030  (-0.03, -0.02) | < 0.001 | -0.342  (-0.36, -0.32) | < 0.001 | .083  (0.07, 0.09) | < 0.001 |
|  | **Total** | -0.033  (-0.20, 0.07) | < 0.001 | -0.320  (-0.33, -0.30) | < 0.001 | 0.076  (-0.13, 0.31) | < 0.001 |
|  | **Urban** | -.023  (-.02, -.01) | < 0.001 | -0.375  (-0.39, -0.36) | < 0.001 | .054  (.04, .06) | < 0.001 |
|  | **Rural** | -.062  (-.06, -.05) | < 0.001 | -0.171  (-0.19, -0.14) | < 0.001 | .130  (.12, .14) | < 0.001 |
| **performed HbA1C [n]** | **Female** | -0.020  (-0.02, -0.01) | < 0.001 | -0.395  (-0.41, -0.37) | < 0.001 | -0.008  (-0.01, 0.001) | < 0.001 |
|  | **Male** | -0.009  (-0.01, -0.002) | < 0.001 | -0.490  (-0.51, -0.46) | < 0.001 | 0.066  (0.05, 0.07) | < 0.001 |
|  | **Total** | -0.016  (-0.22, 0.12) | < 0.001 | -0.432  (-0.44, -0.41) | < 0.001 | 0.022  (-0.25, 0.33) | < 0.001 |
|  | **Urban** | -.017  (-.02, -.01) | < 0.001 | -0.476  (-0.49, -0.45) | < 0.001 | .012  (.003, .02) | 0.005 |
|  | **Rural** | -.010  (-.02, -.0003) | < 0.001 | -0.269  (-0.30, -0.23) | < 0.001 | .051  (.03, .06) | < 0.001 |

**Table S12** Interrupted time series analysis in patient with type 2 diabetes by optimal and suboptimal BMI during 2019-2020

| **Variable** | | **Trend before COVID-19 pandemic** | | **Level changed by COVID-19 natural intervention** | | **Recovery (trend) during COVID-19 pandemic** | |
| --- | --- | --- | --- | --- | --- | --- | --- |
|  |  | **β1**  **(95% CI)** | **p-value** | **β2**  **(95% CI)** | **p-value** | **β3**  **(95% CI)** | **p-value** |
| **BMI < 25 [%]** | Female | .027  (-.14, .19) | 0.745 | .007  (-0.53, 0.55) | 0.980 | -.037  (-.27, .19) | 0.751 |
|  | Male | .032  (-.12, .19) | 0.691 | -0.010  (-0.52, 0.50) | 0.969 | -.058  (-.28, .16) | 0.607 |
|  | Total | .029  (-.13, .19) | 0.727 | .0007  (-0.53, 0.53) | 0.998 | -.045  (-.27, .18) | 0.701 |
|  | Urban | .036  (-.13, .20) | 0.670 | 0.021  (-0.52, 0.57) | 0.940 | -.052  (-.28, .18) | 0.664 |
|  | Rural | .017  (-.13, .17) | 0.828 | -0.049  (-0.56, 0.46) | 0.849 | -.035  (-.25, .18) | 0.755 |
| **BMI 25-30 [%]** | Female | -.006  (-.13, .11) | 0.922 | -0.019  (-0.43, 0.39) | 0.927 | .019  (-.15, .19) | 0.826 |
|  | Male | -.011  (-.13, .11) | 0.862 | 0.005  (-0.41, 0.42) | 0.978 | .027  (-.15, .20( | 0.763 |
|  | Total | -.007  (-.13, .11) | 0.904 | -.010  (-0.42, 0.40) | 0.962 | .022  (-.15, .19) | 0.804 |
|  | Urban | -.015  (-.13, .10) | 0.808 | -0.026  (-0.44, 0.38) | 0.902 | .040  (-.13, .21) | 0.654 |
|  | rural | .007  (-.12, .13) | 0.909 | 0.018  (-0.40, 0.43) | 0.933 | -.008  (-.18, .17) | 0.922 |
| **BMI ≥ 30 [%]** | Female | -.019  (-.20, .16) | 0.835 | 0.029  (-0.57, 0.63) | 0.924 | .002  (-.25, .26) | 0.984 |
|  | Male | -.020  (-.21, .17) | 0.834 | -0.005  (-0.64, 0.63) | 0.986 | .020  (-.25, .29) | 0.881 |
|  | Total | -.019  (-.20, .16) | 0.836 | 0.017  (-0.60, 0.63) | 0.956 | .007  (-.25, .27) | 0.953 |
|  | Urban | -.010  (-.19, .17) | 0.911 | 0.027  (-0.57, 0.63) | 0.929 | -.025  (-.28, .23) | 0.851 |
|  | Rural | -.042  (-.23, .14) | 0.661 | 0.035  (-0.61, 0.68) | 0.914 | .072  (-.19, .34) | 0.599 |

**Table S13** Interrupted time series analysis in patient with type 2 diabetes by FBS during 2019-2020

| **Variable** | | **Trend before COVID-19 pandemic** | | **Level changed by COVID-19 natural intervention** | | **Recovery (trend) during COVID-19 pandemic** | |
| --- | --- | --- | --- | --- | --- | --- | --- |
|  |  | **β1**  **(95% CI)** | **p-value** | **β2**  **(95% CI)** | **p-value** | **β3**  **(95% CI)** | **p-value** |
| **FBS < 70 [%]** | Female | .024  (-.24, .29) | 0.859 | 0.156  (-0.69, 1.01) | 0.720 | -.074  (-.43, .29) | 0.691 |
|  | Male | .0007  (-.24, .24) | 0.995 | -0.007  (-0.82, 0.80) | 0.986 | .003  (-.34, .35) | 0.982 |
|  | Total | .013  (-.24, .27) | 0.921 | 0.099  (-0.73, 0.93) | 0.816 | -.041  (-.39, .31) | 0.819 |
|  | Urban | .065  (-.18, .31) | 0.606 | 0.017  (-0.77, 0.81) | 0.965 | -.106  (-.44, .23) | 0.545 |
|  | Rural | -.190  (-.48, .10) | 0.207 | 0.485  (-0.54, 1.51) | 0.354 | .213  (-.19, .62) | 0.312 |
| **FBS 70-130 [%]** | Female | .011  (-.10, .12) | 0.849 | -.050  (-0.43, 0.33) | 0.799 | .004  (-.16, .16) | 0.953 |
|  | Male | .005  (-.11, .12) | 0.929 | -0.036  (-0.42, 0.35) | 0.854 | .017  (-.14, .18) | 0.833 |
|  | Total | .009  (-.10, .12) | 0.874 | -0.045  (-0.43, 0.34) | 0.816 | .009  (-.15, .17) | 0.910 |
|  | Urban | .004  (-.11, .12) | 0.934 | -0.035  (-0.41, 0.34) | 0.857 | .012  (-.15, .17) | 0.876 |
|  | Rural | .018  (-.10, .13) | 0.761 | -0.059  (-0.45, 0.33) | 0.771 | .002  (-.16, .17) | 0.976 |
| **FBS ≥ 130 [%]** | Female | -.027  (-.18, .12) | 0.726 | 0.028  (-0.48, 0.54) | 0.915 | .017  (-.20, .23) | 0.879 |
|  | Male | -.010  (-.16, .14) | 0.898 | 0.068  (-0.45, 0.58) | 0.797 | -.032  (-.25, .18) | 0.773 |
|  | Total | -.021  (-.17, .13) | 0.786 | 0.043  (-0.47, 0.56) | 0.869 | -.001  (-.22, .21) | 0.989 |
|  | Urban | -.036  (-.19, .12) | 0.658 | 0.055  (-0.48, 0.59) | 0.840 | .018  (-.21, .24) | 0.872 |
|  | Rural | .018  (-.12, .16) | 0.797 | -0.027  (-0.49, 0.44) | 0.909 | -.055  (-.25, .14) | 0.590 |

**Table S14** Interrupted time series analysis in patient with type 2 diabetes by HbA1C during 2019-2020

| **Variable** | | **Trend before COVID-19 pandemic** | | **Level changed by COVID-19 natural intervention** | | **Recovery (trend) during COVID-19 pandemic** | |
| --- | --- | --- | --- | --- | --- | --- | --- |
|  |  | **β1**  **(95% CI)** | **p-value** | **β2**  **(95% CI)** | **p-value** | **β3**  **(95% CI)** | **p-value** |
| **HbA1C < 7 [%]** | Female | .032  (-.11, .17) | 0.663 | -0.170  (-0.65, 0.31) | 0.491 | .013  (-.19, .22) | 0.899 |
|  | Male | .010  (-.13, .15) | 0.889 | -0.054  (-0.54, 0.43) | 0.827 | .010  (-.19, .21) | 0.924 |
|  | Total | .023  (-.12, .17) | 0.751 | -0.125  (-0.61, 0.36) | 0.614 | .010  (-.19و .21) | 0.919 |
|  | Urban | .037  (-.1093, .18) | 0.619 | -0.164  (-0.64, 0.32) | 0.505 | .010  (-.19, .218) | 0.918 |
|  | Rural | -.033  (-.18, .11) | 0.657 | 0.040  (-0.45, 0.53) | 0.872 | .028  (-.18, .24) | 0.788 |
| **HbA1C 7-7.5 [%]** | Female | .006  (-.15, .16) | 0.940 | 0.151  (-0.37, 0.68) | 0.574 | -.046  (-.27و .17( | 0.685 |
|  | Male | .039  (-.11, .19) | 0.627 | 0.003  (-0.50, 0.51) | 0.988 | -.068  (-.29, .15) | 0.541 |
|  | Total | .020  (-.14, .18) | 0.808 | 0.092  (-0.43, 0.61) | 0.730 | -.056  (-.27, .16) | 0.623 |
|  | Urban | .023  (-.13, .18) | 0.774 | 0.117  (-0.39, 0.63) | 0.656 | -.072  (-.29, .14) | 0.521 |
|  | Rural | .005  (-.15, .17) | 0.946 | 0.026  (-0.51, 0.57) | 0.923 | .003  (-.22, .23) | 0.975 |
| **HbA1C 7.5-8 [%]** | Female | -.029  (-.22, .17) | 0.775 | -0.007  (-0.68, 0.66) | 0.982 | .023  (-.26, .31) | 0.874 |
|  | Male | -.036  (-.24, .16) | 0.730 | 0.048  (-0.64, 0.74) | 0.890 | .035  (-.25, .32) | 0.812 |
|  | Total | -.030  (-.23, .17) | 0.765 | 0.010  (-0.67, 0.69) | 0.976 | .026  (-.26, .31) | 0.857 |
|  | Urban | -.035  (-.23, .16) | 0.728 | 0.002  (-0.67, 0.68) | 0.994 | .023  (-.26, .31) | 0.872 |
|  | Rural | -.012  (-.22, .19) | 0.907 | 0.041  (-0.64, 0.73) | 0.907 | .027  (-.26, .31) | 0.853 |
| **HbA1C 8-8.5 [%]** | Female | -.012  (-.29, .27) | 0.930 | -0.071  (-1.03, 0.89) | 0.884 | .032  (-.37, .44) | 0.878 |
|  | Male | -.055  (-.34, .23) | 0.705 | 0.070  (-0.90, 1.04) | 0.887 | .057  (-.35, .46) | 0.784 |
|  | Total | -.026  (-.31, .25) | 0.856 | -0.020  (-0.98, 0.94) | 0.967 | .036  (-.37, .44) | 0.861 |
|  | Urban | -.071  (-.36, .21) | 0.630 | 0.088  (-0.91, 1.08) | 0.863 | .066  (-.35, .48) | 0.757 |
|  | Rural | .121  (-.14, .39) | 0.378 | -0.385  (-1.25, 0.48) | 0.387 | -.075  (-.45, .30) | 0.700 |
| **HbA1C ≥ 8.5 [%]** | Female | -.104  (-.45, .24) | 0.552 | 0.314  (-0.85, 1.48) | 0.600 | .047  (-.44, .53) | 0.851 |
|  | Male | -.060  (-.40, .28) | 0.729 | 0.023  (-1.14, 1.19) | 0.968 | .098  (-.39, .58) | 0.692 |
|  | Total | -.085  (-.42, .25) | 0.625 | 0.190  (-0.98, 1.36) | 0.749 | .072  (-.41, .56) | 0.771 |
|  | Urban | -.114  (-.46965 .24) | 0.529 | 0.176  (-1.05, 1.41) | 0.779 | .135  (-.37, .64) | 0.604 |
|  | Rural | -.004  (-.31, .30) | 0.975 | 0.149  (-0.85, 1.15) | 0.770 | -.108  (-.54, .32) | 0.627 |

**Table S15** Interrupted time series analysis in patient with type 2 diabetes by HTN and HTN well control during 2019-2020

| **Variable** | | **Trend before COVID-19 pandemic** | | **Level changed by COVID-19 natural intervention** | | **Recovery (trend) during COVID-19 pandemic** | |
| --- | --- | --- | --- | --- | --- | --- | --- |
|  |  | **β1**  **(95% CI)** | **p-value** | **β2**  **(95% CI)** | **p-value** | **β3**  **(95% CI)** | **p-value** |
| **Diabetic with HTN [n]** | Female | -.022  (-.02, -.01) | < 0.001 | -0.430  (-0.45, -0.40) | < 0.001 | .072  (.06, .08) | < 0.001 |
|  | Male | -.031  (-.04, -.02) | < 0.001 | -0.413  (-0.44, -0.38) | < 0.001 | .083  (.06, .09) | < 0.001 |
|  | Total | -.025  (-.03, -.02) | < 0.001 | -0.424  (-0.44, -0.40) | < 0.001 | .076  (.06, .08) | < 0.001 |
|  | Urban | -.039  (-.04, -.03) | < 0.001 | -0.483  (-0.50, -0.45) | < 0.001 | .095  (.08, .10) | < 0.001 |
|  | rural | .003  (-.006, .01) | 0.492 | -0.322  (-0.35, -0.28) | < 0.001 | .039  (.02, .05) | < 0.001 |
| **Diabetic with HTN well control [%]** | Female | -.097  (-.33, .13) | 0.419 | -0.349  (-1.23, 0.53) | 0.440 | .207  (-.16, .58) | 0.279 |
|  | Male | -.098  (-.34, .14) | 0.427 | -0.271  (-1.17, 0.62) | 0.554 | .196  (-.18, .57) | 0.312 |
|  | Total | -.099  (-.33, .13) | 0.414 | -0.312  (-1.20, 0.57) | 0.492 | .202  (-.17, .57) | 0.294 |
|  | Urban | -.084  (-.34, .17) | 0.523 | -0.437  (-1.42, 0.54) | 0.384 | .180  (-.24, .60) | 0.401 |
|  | Rural | -.120  (-.31, .06) | 0.211 | -0.061  (-0.74, 0.62) | 0.860 | .216  (-.06, .50) | 0.135 |

**Table S16** Interrupted time series analysis in patient with type 2 diabetes by new Complication, refer to level 2 and emergency refer during 2019-2020

| **Variable** | | **Trend before COVID-19 pandemic** | | **Level changed by COVID-19 natural intervention** | | **Recovery (trend) during COVID-19 pandemic** | |
| --- | --- | --- | --- | --- | --- | --- | --- |
|  |  | **β1**  **(95% CI)** | **p-value** | **β2**  **(95% CI)** | **p-value** | **β3**  **(95% CI)** | **p-value** |
| **new Complication of T2D [n]** | Female | .061  (.02, .10) | 0.002 | -0.539  (-0.67, 0.40) | < 0.001 | -.116  (-.17, -.05) | < 0.001 |
|  | Male | .122  (.07, .16) | < 0.001 | -0.603  (-0.75, -0.45) | < 0.001 | -.189  (-.25, -.11) | < 0.001 |
|  | Total | .087  (.05, .11) | < 0.001 | -0.566  (-0.66, -0.46) | < 0.001 | -.147  (-.19, -.10) | < 0.001 |
|  | Urban | .097  (.06, .12) | < 0.001 | -0.639  (-0.75, -0.52) | < 0.001 | -.23  (-.28, -.17) | < 0.001 |
|  | Rural | .042  (-.02, .11) | 0.239 | -0.220  (-0.45, 0.01) | 0.062 | .082  (-.01, .17) | 0.099 |
| **Refer to level 2 [n]** | Female | .249  (.23, .26) | < 0.001 | -1.01  (-1.05, -0.97) | < 0.001 | -.106  (-.12, -.08) | < 0.001 |
|  | Male | .258  (.24, .27) | < 0.001 | -0.964  (-1.009, -0.91) | < 0.001 | -.123  (-.14, -.10) | < 0.001 |
|  | Total | .252  (.24, .26) | < 0.001 | -0.994  (-1.02, -0.96) | < 0.001 | -.113  (-.12, -.10) | < 0.001 |
|  | Urban | .318  (.3079, .32) | < 0.001 | -1.14  (-1.17, -1.11) | < 0.001 | -.168  (-.18, -.15) | < 0.001 |
|  | Rural | .038  (.02, .05) | < 0.001 | -0.468  (-0.53, -0.40) | < 0.001 | .065  (.03, .09) | < 0.001 |
| **Emergency refer [n]** | Female | .054  (.02, .08) | < 0.001 | -0.781  (-0.88, -0.67) | < 0.001 | .150  (.10, .19) | < 0.001 |
|  | Male | -.060  (-.09, -.02) | 0.001 | 0.046  (-0.07, 0.16) | 0.441 | .073  (.02, .12) | 0.004 |
|  | Total | .005  (-.01, .02) | 0.608 | -0.401  (-0.48, -0.32) | < 0.001 | .106  (.07, .13) | < 0.001 |
|  | Urban | .059  (.03, .08) | < 0.001 | -0.537  (-0.62, -0.45) | < 0.001 | .035  (-.001, .07) | 0.063 |
|  | Rural | -.239  (-.29, -.18) | < 0.001 | 0.269  (0.07, 0.46) | 0.007 | .417  (.33, .49) | < 0.001 |

**Table S17** Interrupted time series analysis in patient with type 2 diabetes by do not go to the health centers due to death, migrate and other reasons during 2019-2020

| **Variable** | | **Trend before COVID-19 pandemic** | | **Level changed by COVID-19 natural intervention** | | **Recovery (trend) during COVID-19 pandemic** | |
| --- | --- | --- | --- | --- | --- | --- | --- |
|  |  | **β1**  **(95% CI)** | **p-value** | **β2**  **(95% CI)** | **p-value** | **β3**  **(95% CI)** | **p-value** |
| **Do not go to the health centers due to death [n]** | Female | .127  (.02, .22) | 0.013 | -0.083  (-0.39, 0.22) | 0.598 | -.088  (-.22, .04) | 0.194 |
|  | Male | .017  (-.09, .12) | 0.755 | 0.257  (-0.09, 0.60) | 0.150 | .017  (-.12, .16) | 0.815 |
|  | Total | .078  (.003, .15) | 0.04 | 0.069  (-0.16, 0.30) | 0.559 | -.040  (-.13, .05) | 0.415 |
|  | Urban | .122  (.02, .22) | 0.015 | -0.127  (-0.43, 0.17) | 0.413 | -.071  (-.20, .05) | 0.283 |
|  | Rural | .017  (-.09, .13) | 0.769 | 0.334  (-0.02, 0.69) | 0.068 | .001  (-.14, .14) | 0.988 |
| **Do not go to the health centers due to migrate [n]** | Female | .148  (.08, .21) | < 0.001 | -0.399  (-0.59, 0.20) | < 0.001 | -.151  (-.24, -.06) | 0.001 |
|  | Male | .022  (-.05, .10) | 0.578 | 0.258  (0.01, 0.50) | 0.040 | -.084  (-.18, .02) | 0.115 |
|  | Total | .099  (.05, .14) | < 0.001 | -0.131  (-0.28, 0.02) | 0.095 | -.128  (-.19, -.06) | < 0.001 |
|  | Urban | .131  (.07, .18) | < 0.001 | -0.232  (-0.41, -0.05) | 0.011 | -.120  (-.19, -.04) | 0.003 |
|  | Rural | .010  (-.08, .10) | 0.829 | 0.158  (-0.14, 0.46) | 0.308 | -.169  (-.30, -.03) | 0.014 |
| **Do not go to the health centers due to other [n]** | Female | .198  (.19, .20) | < 0.001 | -0.074  (-0.08, -0.06) | < 0.001 | -.215  (-.22, -.20) | < 0.001 |
|  | Male | .201  (.19, .20) | < 0.001 | -0.161  (-0.17, -0.14) | < 0.001 | -.188  (-.19, -.18) | < 0.001 |
|  | Total | .200  (.19, .20) | < 0.001 | -0.109  (-0.11, -0.09) | < 0.001 | -.204  (-.20, -.20) | < 0.001 |
|  | Urban | .186  (.18, .18) | < 0.001 | -0.099  (-0.10, -0.08) | < 0.001 | -.184  (-.18, -.18) | < 0.001 |
|  | Rural | .360  (.34, .37) | < 0.001 | -0.246  (-0.27, -0.21) | < 0.001 | -.429  (-.44, -.41) | < 0.001 |

Table S18 Correlation of seasonal T2DM care numbers and seasonal rates of the COVID-19 incidence rate, hospitalization and mortality rate during the first year of the COVID-19 pandemic in Fars province, Southern Iran.

| Index | Infection rate ^2^ | Hospital admission rate ^3^ | Mortality rate ^4^ | |
| --- | --- | --- | --- | --- |
| Health worker visit | 0.400, 0.600^1^ | -0.200, 0.800 | 0.400, 0.600 | |
| Physician visit | 0.400, 0.600 | 0.000, 1.000 | 0.400, 0.600 | |
| BMI assessment | 0.400, 0.600 | -0.200, 0.800 | 0.400, 0.600 | |
| FBS measurement | 0.400, 0.600 | 0.000, 1.000 | 0.400, 0.600 | |
| HbA1C measurement | 0.800, 0.200 | 0.600, 0.400 | 0.800, 0.200 | |
| BMI <25 kg/m^2^ | 0.200, 0.800 | -0.400, 0.600 | 0.200, 0.800 | |
| FBS 70-130 mg/dL | 0.400, 0.600 | 0.000, 1.000 | 0.400, 0.600 | |
| HbA1c <7% | 0.400, 0.600 | 0.000. 1.000 | 0.400, 0.600 | |
| Patients with T2DM with controlled HTN | 0.400, 0.600 | -0.200, 0.800 | 0.400, 0.600 | |
| Newly diagnosed diabetes complications [n (per 1000)] | -0.400, 0.600 | -0.200, 0.800 | -0.400, 0.600 | |
| Refer to hospital emergency ward [n (per 1000] | 0.200, 0.800 | -0.400, 0.600 | 0.200, 0.800 | |
| Do not go to the health centers because of death [n (per 10000)] | 1.000, <0.0001 | 0.800, 0.200 | 1.000, <0.0001 | |
| Do not go to the health centers because of other reasons [n (%)] | 0.800, 0.200 | 1.000, <0.0001 | 0.800, 0.200 | |
| ^1^ Spearman’s rho [coefficient, *P*]  ^2^ Counts: 9,209; 39,783; 108,704; 23,171.  ^3^ Counts: 10,371; 14,946; 21,703; 7,970.  ^4^ Counts: 123; 792; 1,987; 416. | | | |  |
