# Supplementary material for: The effect of COVID-19 pandemic on diabetes care indices in Southern Iran: an interrupted time series analysis
Source: BMC Health Serv Res. 2023 Feb 13;23:148. doi: 10.1186/s12913-023-09158-4 (PMC9925215; doi:10.1186/s12913-023-09158-4)
Supplement: Supplementary file 2 — Additional file 2: Figure S1. Prevalence of T2DM in female during 2019-2020. Figure S2. Prevalence of T2DM in male during 2019-2020. Figure S3. Incidence rate of T2DM in female during 2019-2020. Figure S4. Incidence rate of T2DM in male during 2019-2020. Figure S5. Number of performed visit health worker in female during 2019-2020. Figure S6. Number of performed visit health worker in male during 2019-2020. Figure S7. Number of performed visit doctor in female during 2019-2020. Figure S8. Number of performed visit doctor in male during 2019-2020. Figure S9. Number of measured BMI in female during 2019-2020. Figure S10. Number of measured BMI in male during 2019-2020. Figure S11. Number of performed FBS in female during 2019-2020. Figure S12. Number of performed FBS in male during 2019-2020. Figure S13. Number of performed HbA1C in female during 2019-2020. Figure S14. Number of performed HbA1C in male during 2019-2020. Figure S15. Percent of BMI < 25 in patient with T2DM during 2019-2020. Figure S16. Percent of BMI 25-30 in patient with T2DM during 2019-2020. Figure S17. Percent of BMI ≥ 30 in patient with T2DM during 2019-2020. Figure S18. Percent of FBS < 70 in patient with T2DM during 2019-2020. Figure S19. Percent of FBS 70-130 in patient with T2DM during 2019-2020. Figure S20. Percent of FBS ≥ 130 in patient with T2DM during 2019-2020. Figure S21. Percent of HbA1C < 7 in patient with T2DM during 2019-2020. Figure S22. Percent of HbA1C 7-7.5 in patient with T2DM during 2019-2020. Figure S23. Percent of HbA1C 7.5-8 in patient with T2DM during 2019-2020. Figure S24. Percent of HbA1C 8-8.5 in patient with T2DM during 2019-2020. Figure S25. Percent of HbA1C ≥ 8.5 in patient with T2DM during 2019-2020. Figure S26. Number of diabetics with HTN in patient with T2DM during 2019-2020. Figure S27. Percent of diabetics with HTN well control in patient with T2DM during 2019-2020. Figure S28. Number of new complications of diabetes in patient with T2DM during 2019-2020. Figur [file 12913_2023_9158_MOESM2_ESM.docx]

**Figure S1** Prevalence of T2DM in female during 2019-2020

**Figure S2** Prevalence of T2DM in male during 2019-2020

**Figure S3** Incidence rate of T2DM in female during 2019-2020

**Figure S4** Incidence rate of T2DM in male during 2019-2020

**Figure S5** Number of performed visit health worker in female during 2019-2020

**Figure S6** Number of performed visit health worker in male during 2019-2020

**Figure S7** Number of performed visit doctor in female during 2019-2020

**Figure S8** Number of performed visit doctor in male during 2019-2020

**Figure S9** Number of measured BMI in female during 2019-2020

**Figure S10** Number of measured BMI in male during 2019-2020

**Figure S11** Number of performed FBS in female during 2019-2020

**Figure S12** Number of performed FBS in male during 2019-2020

**Figure S13** Number of performed HbA1C in female during 2019-2020

**Figure S14** Number of performed HbA1C in male during 2019-2020

**Figure S15** Percent of BMI < 25 in patient with T2DM during 2019-2020

**Figure S16** Percent of BMI 25-30 in patient with T2DM during 2019-2020

**Figure S17** Percent of BMI ≥ 30 in patient with T2DM during 2019-2020

**Figure S18** Percent of FBS < 70 in patient with T2DM during 2019-2020

**Figure S19** Percent of FBS 70-130 in patient with T2DM during 2019-2020

**Figure S20** Percent of FBS ≥ 130 in patient with T2DM during 2019-2020

**Figure S21** Percent of HbA1C < 7 in patient with T2DM during 2019-2020

**Figure S22** Percent of HbA1C 7-7.5 in patient with T2DM during 2019-2020

**Figure S23** Percent of HbA1C 7.5-8 in patient with T2DM during 2019-2020

**Figure S24** Percent of HbA1C 8-8.5 in patient with T2DM during 2019-2020

**Figure S25** Percent of HbA1C ≥ 8.5 in patient with T2DM during 2019-2020

**Figure S26** Number of diabetics with HTN in patient with T2DM during 2019-2020

**Figure S27** Percent of diabetics with HTN well control in patient with T2DM during 2019-2020

**Figure S28** Number of new complications of diabetes in patient with T2DM during 2019-2020

**Figure S29** Number of refer to level 2 in patient with T2DM during 2019-2020

**Figure S30** Number of emergency refer in patient with T2DM during 2019-2020

**Figure S31** Number of do not go to the health centers due to death in patient with T2DM during 2019-2020

**Figure S32** Number of do not go to the health centers due to migrate in patient with T2DM during 2019-2020

**Figure S33** Number of do not go to the health centers due to other reasons in patient with T2DM during 2019-2020
